# Single-cell transcriptomics reveals heterogeneous progression and EGFR activation in pancreatic adenosquamous carcinoma

**DOI:** 10.7150/ijbs.58886

**Published:** 2021-06-22

**Authors:** Xin Zhao, Han Li, Shaocheng Lyu, Jialei Zhai, Zhiwei Ji, Zhigang Zhang, Xinxue Zhang, Zhe Liu, Huaguang Wang, Junming Xu, Hua Fan, Jiantao Kou, Lixin Li, Ren Lang, Qiang He

**Affiliations:** 1 Department of Hepatobiliary Surgery, Beijing Chaoyang Hospital affiliated to Capital Medical University, Beijing 100020, China.; 2 Department of Head and Neck Surgery, National Cancer Center/National Clinical Research Center for Cancer/Cancer Hospital, Chinese Academy of Medical Sciences and Peking Union Medical College, Beijing, 100021, China.; 3 Department of Pathology, Beijing Chaoyang Hospital affiliated to Capital Medical University, Beijing 100020, China.; 4 College of Artificial Intelligence, Nanjing Agricultural University, Nanjing, Jiangsu 210095, China.; 5 School of Information Management and Statistics, Hubei University of Economics, Wuhan 430205, Hubei, China.; 6 Department of Pharmacology, Beijing Chaoyang Hospital affiliated to Capital Medical University, Beijing 100020, China.

**Keywords:** pancreatic adenosquamous carcinoma, single-cell RNA sequencing, intraductal papillary mucinous neoplasm, heterogeneity, cell-cell communication, pancreatic cancer

## Abstract

Pancreatic adenosquamous carcinoma (PASC) — a rare pathological pancreatic cancer (PC) type — has a poor prognosis due to high malignancy. To examine the heterogeneity of PASC, we performed single-cell RNA sequencing (scRNA-seq) profiling with sample tissues from a healthy donor pancreas, an intraductal papillary mucinous neoplasm, and a patient with PASC. Of 9,887 individual cells, ten cell subpopulations were identified, including myeloid, immune, ductal, fibroblast, acinar, stellate, endothelial, and cancer cells. Cancer cells were divided into five clusters. Notably, cluster 1 exhibited stem-like phenotypes expressing UBE2C, ASPM, and TOP2A. We found that S100A2 is a potential biomarker for cancer cells. LGALS1, NPM1, RACK1, and PERP were upregulated from ductal to cancer cells. Furthermore, the copy number variations in ductal and cancer cells were greater than in the reference cells. The expression of EREG, FCGR2A, CCL4L2, and CTSC increased in myeloid cells from the normal pancreas to PASC. The gene sets expressed by cancer-associated fibroblasts were enriched in the immunosuppressive pathways. We demonstrate that EGFR-associated ligand-receptor pairs are activated in ductal-stromal cell communications. Hence, this study revealed the heterogeneous variations of ductal and stromal cells, defined cancer-associated signaling pathways, and deciphered intercellular interactions following PASC progression.

## Introduction

Pancreatic cancer (PC) is the most lethal gastrointestinal cancer in humans, with a five-year survival rate of 7% 1. Pancreatic adenosquamous carcinoma (PASC) is a mixed pancreatic malignant tumor comprising adenocarcinoma and squamous cell carcinoma in histology, accounting for 1%-4.4% of PCs 2, 3. Notably, PASC is highly aggressive and its prognosis is poor, with a two-year survival rate of 13% 2. Due to the low incidence, the tumorigenesis, progression, and molecular characteristics of PASC remains unclear and thus, there is no molecular target therapy for PASC.

The identification of molecular biomarkers and malignancy-driving signaling pathways are important to better understand the regulatory mechanisms of tumor progression and to develop potential therapeutic targets for PASC. Genomic and transcriptomic studies have discovered the mutations of TP53 and KRAS2 and the loss of p16 protein in PASC 4, 5. Currently, single-cell RNA sequencing (scRNA-seq) aids in the profiling and clustering of multiple cell types in tumor tissues. Thus, tumor heterogeneity can be delineated to identify new biomarkers and targets, especially for rare cancer types. A previous study revealed progressive T cell depletion and stromal myofibroblast activation in pancreatic neoplasms during the progression of intraductal papillary mucinous neoplasm (IPMN) to pancreatic ductal adenocarcinoma (PDAC) 6. Another study profiled pancreatic malignant cells, revealing intratumoral heterogeneity and critical signaling pathways related to PC improvement 7. However, the heterogeneity of PASC and cell evolution from the healthy pancreas to precancerous lesions and then to PASC remain unclarified.

Here, we performed scRNA-seq in 9,887 cells from the pancreas of a normal individual and tumor tissues of two patients with IPMN and PASC, respectively. Since IPMN is one of the precancerous lesions of PC, we could dissect heterogeneous alterations of ductal and stromal cells along with PASC formation. We identified diverse cell types, including cancer cells, ductal cells, immune cells, myeloid cells, and fibroblasts, based on their unique gene expression patterns. We further discovered the evolutionary features of ductal cells from normal to malignant stages. Finally, we identified several novel genes and signaling pathways that possibly drive PASC progression within ductal and stromal cells. Consequently, our findings may contribute to a thorough understanding of early PASC tumorigenesis events at the single-cell level and provide novel biomarkers and therapeutic targets for PASC.

## Materials and methods

### Patients and fresh tissues

Between March and May 2020, we harvested one normal pancreas, one IPMN, and one PASC sample from the Beijing Chaoyang Hospital. The normal pancreas was from a donor after cardiac death, who donated his liver for transplantation at our hospital. The patient did not suffer from intemperance or chronic pancreatitis. During surgery, we resected the main pancreatic duct along the parenchyma to obtain a large number of pancreatic ductal cells. The patients with IPMN and PASC were diagnosed with pathological findings. The patients' clinical details are presented in Table S1. All patients or donor relatives signed written informed consents. The study was approved by the Ethics Committee (2020-S-274 and 2020-S-302) of the Beijing Chaoyang Hospital.

### Tissue dissociation

Three fresh specimens were stored in GEXSCOPE tissue preservation solution (Singleron Biotechnologies, Nanjing, China), washed three times with Hanks balanced salt solution, and minced into 1-2 mm fragments with a sterile surgical scalpel. Then, the crushed tissue was digested at 37 °C for 15 min in a 15-mL centrifuge tube containing 2 mL GEXSCOPE solution with sustained agitation. The slurry was filtered through a sterile 40 μm cell strainer and centrifuged at 1,000 rpm for 5 min. After discarding the supernatant, the sediment was resuspended in 1 mL PBS (HyClone, Logan, UT, USA). Then, each sample was treated with 2 mL GEXSCOPE red blood cell lysis buffer (Singleron Biotechnologies) for 10 min at 25 °C to remove the red blood cells. The solution was then centrifuged at 500 × g for 5 min and suspended in PBS. Finally, single cells were stained with trypan blue (Sigma, MO, USA) and microscopically evaluated for cell viability.

### Single-cell RNA sequencing

We diluted the single-cell suspensions to 1×10^5^ cells/mL with PBS (HyClone) and loaded them onto microfluidic devices. Next, we used the GEXSCOPE Single-Cell RNA Library Kit (Singleron Biotechnologies) to construct the scRNA-seq libraries 8. Individual libraries were diluted to 4 nM, pooled, and sequenced on an Illumina HiSeq X platform with 150 bp paired-end reads.

### The quantification and statistical analysis for single-cell RNA sequencing

Raw reads were processed to generate gene expression profiles using an internal pipeline (Figure S1). Briefly, after filtering the reads, reads without poly T tails, cell barcodes, and unique molecular identifiers (UMIs) were extracted. Adapters and poly-A tails were trimmed using fastp V1, and the reads were aligned to the GRCh38 human genome assembly and ENSEMBL version 92 gene annotation using STAR 2.5.3a and featureCounts 1.6.2^9^. We grouped the reads with the same cell barcode and then calculated the UMIs per gene per cell.

Violin plots were used to show the number of UMIs, number of genes, and relative mitochondrial and ribosomal transcript abundance. The results suggested that dead cell reads were comparable among samples. Transcript batch effects induced by apoptosis were not observed (Figure S2). Finally, we screened out 9,887 cells that contained more than 300 genes and less than 10% mitochondrial genes for further bioinformatics analysis. Gene expression values were log-normalized, centered, and processed into a matrix.

### Cell type identification

We applied principal component 10 and t-distribution stochastic neighbor embedding (tSNE) analyses to cluster the cells^11^ based on the calculated gene expression matrices. Cell type identification and clustering were performed using the Seurat program (http://satijalab.org/seurat/, R package; v.3.0.1). We set the FindClusters parameter resolution to 0.6 to fulfill cell clustering. Differentially expressed genes (DEGs) were identified using the DESeq2 R package (|log_2_(fold change)| > 1 and *P* < 0.05) and visualized as heatmaps and volcano plots. Based on the DEGs, we used the FindMarkers function to define diverse cell types among different samples or consecutive clusters.

### Validation of the clinical significance of S100A2

Since S100A2 was identified as a marker gene of PASC cells, we further applied public transcriptome databases to explore its clinical significance. We downloaded the GSE28735 dataset from the Gene Expression Omnibus database to validate the DEG expression. This dataset contains mRNA expression profiles of 45-paired PC and adjacent nontumor tissues measured using Affymetrix GeneChip Human Gene 1.0 ST arrays. We then used a paired-sample *t*-test to compare the expression of S100A2 between these two tissue types. We collected S100A2 expression values and the clinical data from the PDAC cohort of The Cancer Genome Atlas (TCGA) database. Based on the median S100A2 value, we classified 186 PC patients into high- and low-level groups and performed Kaplan-Meier survival analyses. Finally, we used the Human Protein Atlas database to examine the S100A2 expression in pancreatic tissues.

### Differential expression analysis for pancreatic adenosquamous carcinoma

We screened out DEGs by performing differential expression analysis between ductal and cancer cells and ranked them by log_2_(fold change) value. Next, we performed Gene Set Enrichment Analysis (GSEA) to evaluate the functions of this gene set using the Molecular Signatures Database v7.2. Three collection sets, including “c5.go.bp.v7.2.entrez.gmt,” “c5.go.cc.v7.2.entrez.gmt,” and “c5.go.mf.v7.2.entrez.gmt” were used to define the Gene Ontology of the biological process, cellular components, and molecular functions for DEGs. We also performed pathway enrichment analysis for DEGs using “c2.cp.wikipathways.v7.2.entrez.gmt” database.

Next, we pooled pancreatic ductal and cancer cells together, clustering five cell subpopulations in ductal cells and four cell types in PASC cells, with principal component analysis and tSNE analysis. Next, we performed differential expression analyses between normal ductal cells and adjacent nonmalignant ductal (ANMD) cells to define a DEG set (|log_2_(fold change)| > 1 and *P* < 0.05). Similarly, another DEG set was determined by comparing the gene expression level between ANMD and PASC cells. Thus, by performing an intersection between these two DEG sets, we could identify the continuously changing genes from normal ductal cells to ANMD and PASC cells.

### Pseudotime analysis

We also used the Monocle2 package (v2.8.0) to deduce the dynamic transcriptome changes within five duct-cell clusters and four cancer cell clusters. Thereby, we could predict the future transcriptional state of an individual cell subgroup. The top ten DEGs (q-value = 0.05) of cluster (C) 1-4 were used to sort PASC cells in a pseudotime order. We further constructed a trajectory tree in a two-dimensional space using the DRTree dimensionality reduction algorithm. Based on pseudotime analysis, we could screen out the upregulated and downregulated genes ranging from ductal cells to PASC cells. After that, we employed the clusterProfiler R package to illuminate the biological processes of DEGs.

### Copy number variation analysis

Based on the scRNA-seq data, we calculated the clonal copy number variation (CNV) in each cell, with a default threshold value of “0.5.” Then, we used the InferCNV R package to map CNVs to DNA locations. After data normalization, we annotated each CNV as either gain or loss, ranging from -1 to +1. We used myeloid and immune cells as reference cells to exclude individual somatic CNVs.

### Cell-cell communication between ductal and stromal cells

We performed cell-cell communication analysis to better understand the interactions between PASC and stromal cells. First, we inputted our scRNA-seq data into CellPhoneDB V2.0 12. In this tool, we calculated the average expression value and the percentage (>10%) of the transcripts encoding the ligands and their receptors in each cell type. Then, we randomly arranged the cell type markers of all cells to form a new cell group and calculated the average expression level of ligands and receptors in this cell group. In this way, a zero distribution was generated for each ligand-receptor pair in each pairwise comparison between two cell types. Thus, we calculated the actual average value of the ligand-receptor pairs between two specific cell types, such as fibroblasts and cancer cells. The significance of the ligand-receptor pair in two cell types was speculated based on the ratio of the actual average value equal to or higher than the average value of the zero distribution. We ranked these ligand-receptor pairs in two specific cell types according to the *P*-values and plot this cell-cell communication network.

### Pathological analyses

Hematoxylin-eosin and immunohistochemical staining were conducted following standard protocols for formalin-fixed paraffin-embedded tissues. We used carcinoma embryonic antigen antibodies to dye IPMN sections and CK8/18 antibodies for normal and PASC sections (Shanghai Shunbai Biotechnology Company; Shanghai, China). From January 2019 to December 2020, we collected five paired PASC and adjacent normal tissues to validate the EGFR expression.

## Results

### scRNA-seq profiles identified ten cell types in the normal pancreas, IPMN, and PASC

**Figure 1:**
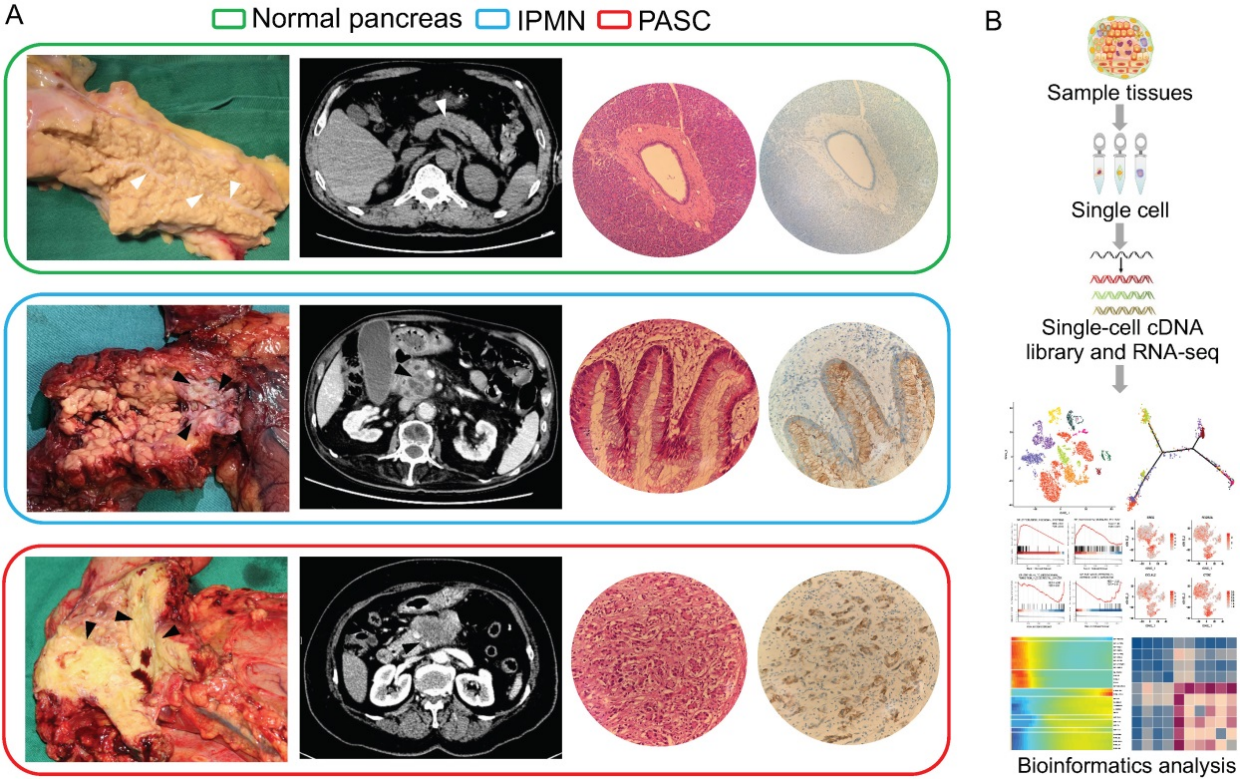
** Clinicopathological manifestations of the normal pancreas, IPMN, and PASC samples.** (**A**) Gross specimens, axial computerized tomography images, hematoxylin-eosin images, and immunohistochemical staining of the normal pancreas (top), IPMN (middle), and PASC (bottom; 200×). White triangles indicate the main pancreatic duct of the normal pancreas, and the black triangles indicate the tumors of IPMN and PASC. (**B**) Single-cell RNA-seq workflow and subsequent bioinformatics analysis.

We used scRNA-seq to investigate the pancreatic heterogeneity ranging from the normal pancreas to those in IPMN and PASC (Figure 1). Within the three histological types, 9,887 cells were sequenced for subsequent analyses. All the scRNA-seq datasets were deposited in the Gene Expression Omnibus site (accession number: GSE165399).

**Figure 2:**
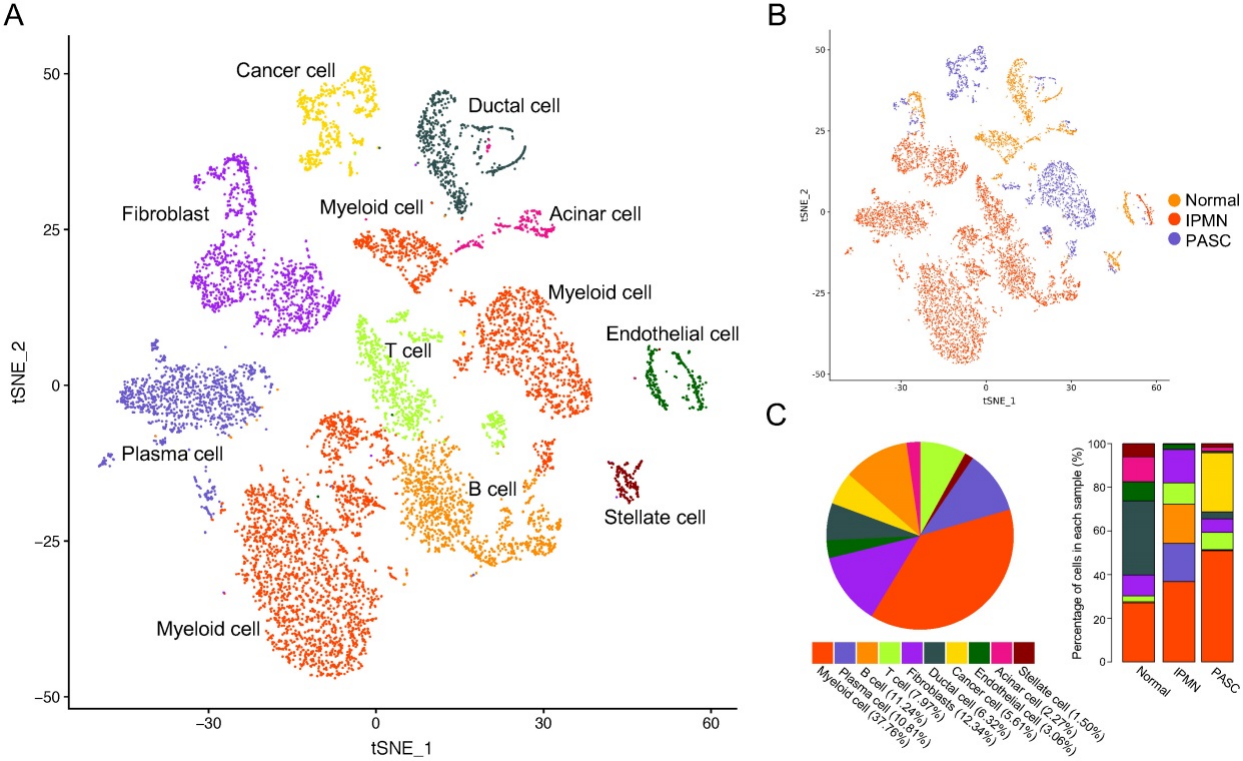
** A total of 9,887 single cells were clustered into ten subgroups based on scRNA-seq.** (**A**) tSNE plot showing ten cell types in tissues of the normal pancreas, IPMN, and PASC. (**B**) Cell distribution across three pancreatic samples. (**C**) Percentage of cell types in the whole (left) and parts of pancreatic specimen (right).

We identified ten cell subpopulations based on gene expression patterns, comprising ductal cells, cancer cells, immune cells, myeloid cells, and fibroblasts (Figure 2A and Table S2). The distribution and percentage of each cell type across the three samples are presented in Figure 2B and C. Myeloid cells were the most dominant cells, accounting for 38.18% (3775/9887) of cell types, and the myeloid cell percentage increased from the healthy pancreas to PASC samples (Figure 2C). Notably, immune cells, including T, B, and plasma cells, were rare in the normal pancreas (3.13%, 52/1662) and PASC samples (8.34%, 173/2074); however, these cells were highly expressed in IPMN (45.15%, 2777/6151). Ductal cells mainly existed in the normal pancreas and were present at low levels in PASC samples.

**Figure 3:**
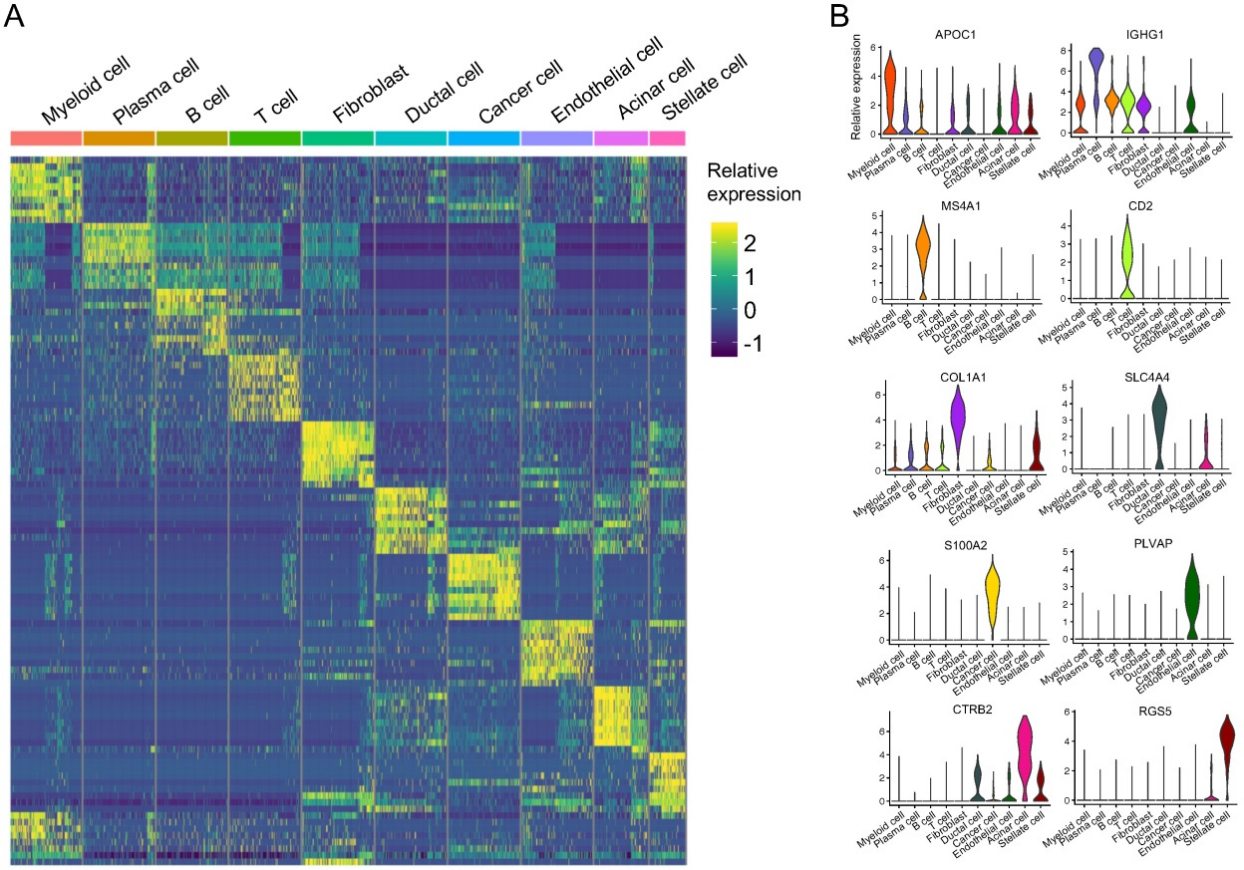
** scRNA-seq demonstrating specific cell subpopulation markers.** (**A**) Heatmap of top ten differentially expressed genes in each cell type. (**B**) Violin plot showing distinct markers of ten cell clusters.

We plotted the top ten DEGs in each cell cluster to highlight their specific identities (Figure 3A). Based on the reported cell markers, we defined the cell clusters as follows: myeloid cells with macrophage markers: IL1B 13 and LYZ 14; plasma cells: IGKC 15 and IGHG2 16; B cells: MS4A1 7 and CD37 17; T cells: CD3D 7 and CD2 18; fibroblasts: COL1A1 19 and LUM 7; ductal cells: CLDN4 20 and CFTR 20; cancer cells: PI3 21; endothelial cells: SPARCL1 22 and CLDN5 23; acinar cells: PRSS1 7 and REG3A 7; and stellate cells: RGS5 7 (Figure 3B and Table S3). Besides SPARCL1, 81% of endothelial cells expressed PLVAP however, only 2% of the other cells expressed PLVAP. The expression value of PLVAP in endothelial cells was significantly higher than that in the other cells (log_10_FC = 2.63 and *P* < 0.001; Table S3). Similarly, S100A2, KRT14, KRT17, and KRT6A were overwhelmingly expressed in cancer cells and their expression values were significantly higher than the other cells (log_10_FC > 3 and *P* < 0.001; Table S3). In addition, CTRB2, REG3A, and REG1A were highly expressed in more than 90% of acinar cells, and CTRB2 was the most different gene compared with the other cells (log_10_FC = 4.67; Table S3). Thus, we identified S100A2, PLVAP, and CTRB2 as novel markers for PASC, endothelial, and acinar cells, respectively (Figure 3B).

Consequently, we applied public databases to reveal the clinical significance of S100A2, a PASC cell gene marker. In the GSE28735 dataset, S100A2 was highly expressed in PC tissues compared to adjacent normal tissues. Furthermore, S100A2 was correlated with poor prognosis in the PDAC cohort of the TCGA dataset. In Human Protein Atlas, S100A2 protein was also overexpressed in PDAC tumors but was not detected in the normal pancreas (Figure S3). These findings suggest that S100A2 is associated with the poor clinical features of PASC.

### Ribosomal hyperfunction is associated with PASC cells

**Figure 4:**
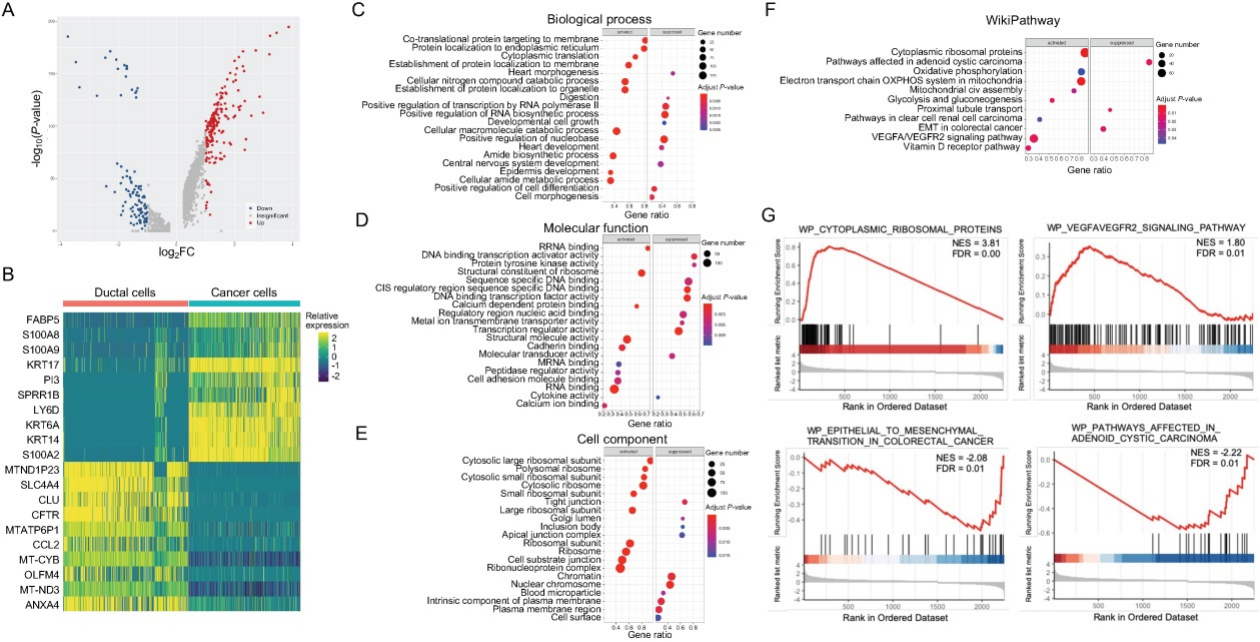
** Functions of DEGs between pancreatic ductal and PASC cells.** (**A**) and (**B**) Volcano plot and heatmap showing the DEGs of PASC cells. (**C**-**E**) Gene Ontology annotations for DEGs based on GSEA. (**F**) Pathway enrichment analyses for DEGs based on WikiPathways gene sets. (**G**) In cancer cells, upregulated genes were associated with “cytoplasmic ribosomal proteins” and “VEGFA/VEGFR2” signaling pathways, while downregulated genes were correlated with “EMT” and “adenoid cystic carcinoma” pathways.

To elucidate the abnormal cellular functions during PASC progression, we compared the expression of 2,350 genes between cancer and ductal cells, identifying DEGs (Figure 4A and B). Gene Ontology analyses showed that this gene set was significantly associated with the activation of “cotranslational protein targeting to membrane” and the inactivation of “positive regulation of RNA biosynthetic process” in biological processes (Figure 4C). In molecular function, these DEGs mainly participated in the upregulation of "structural constituent of ribosomes" and the downregulation of “DNA binding and transcription activator activity” (Figure 4D). The dysregulated genes also correlated with tumor-promoting functions in cellular components, such as the activation of “ribosomal subunits” and the suppression of “tight junctions” (Figure 4E). We further observed that the DEGs were enriched in the activation of “cytoplasmic ribosomal proteins” and “VEGFA/VEGFR2 signaling pathway,” according to the WikiPathways dataset (Figure 4F and G). In contrast, this gene set correlated with the inactivation of “epithelial-mesenchymal transition (EMT)” and “adenoid cystic carcinoma” (Figure 4F and G).

### LGALS1, NPM1, RACK1, and PERP are overexpressed from ductal to PASC cells

**Figure 5:**
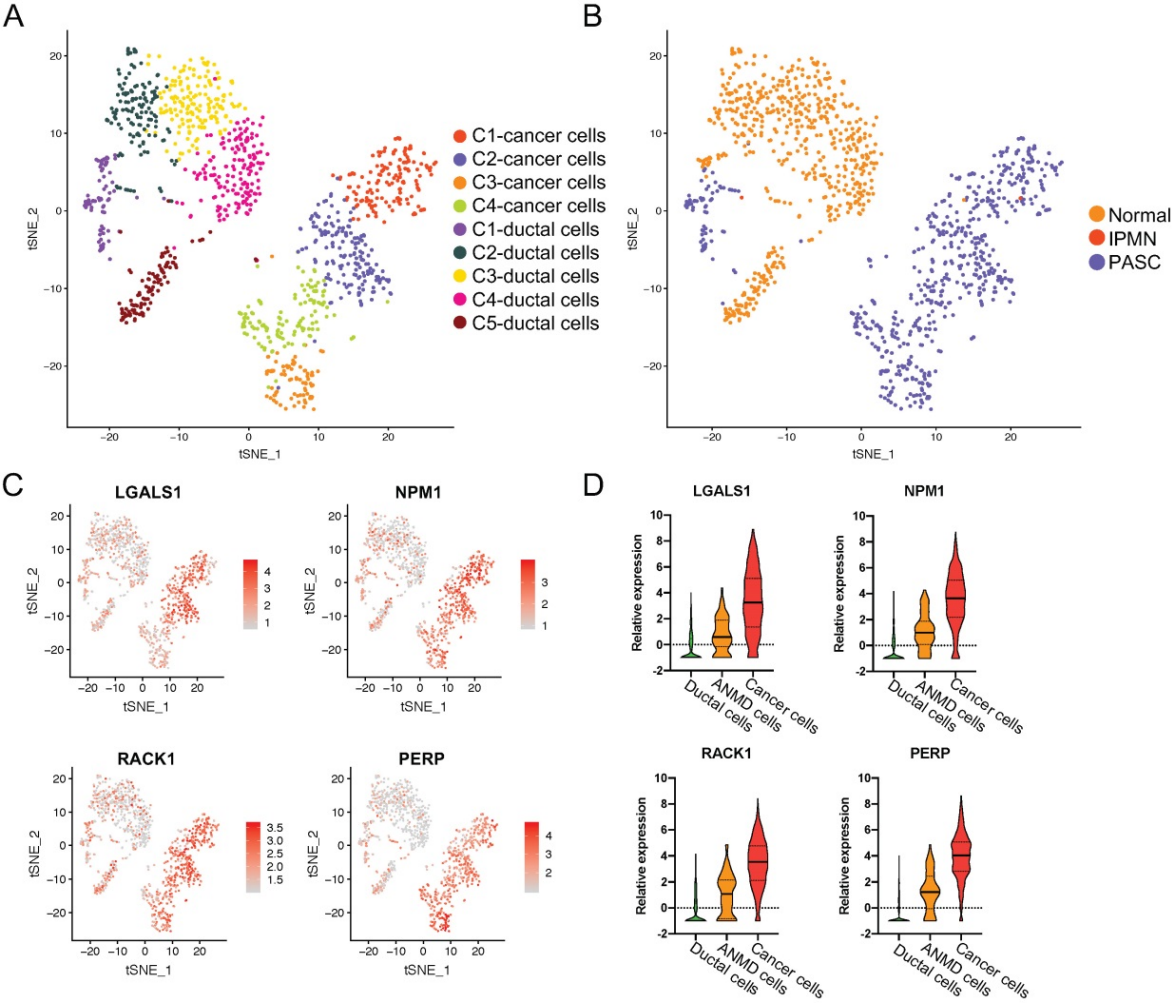
** The upregulated genes across ductal, adjacent nonmalignant, and PASC cells.** (**A**) tSNE plot showing four cancer cell clusters and five duct-cell clusters. (**B**) Total cell distribution suggesting that C1 of ductal cells is derived from the adjacent normal tissue in PASC. (**C**) tSNE plot showing the expression level and distribution of LGALS1, NPM1, RACK1, and PERP in ductal and PASC cells. The red bar indicates relative gene expression level. (**D**) The relative expression levels of LGALS1, NPM1, RACK1, and PERP in ductal, adjacent nonmalignant ductal (ANMD), and cancer cells. Data are presented as mean ± SD.

To investigate the driving factors in PASC tumorigenesis, we pooled ductal and PASC cells together and conducted the principal component analysis using our scRNA-seq dataset. We then divided these into five ductal cell clusters and four carcinoma subgroups (Figure 5A and B). We found that most ductal cells were derived from the normal pancreas (89.08%, 563/632), whereas only 68 ductal cells were derived from the adjacent normal tissue of the PASC sample (Figure 2A and B). Notably, these cells were “entangled,” expressing antitumor genes such as LTF 24, oncogenes such as CXCL6 25, SAA1 26, and S100A9 27, and the inflammation gene CRP. Therefore, we consider this cell type a transitional cell type between healthy ductal and PASC cells. Among the healthy ductal, transitional, and tumor cells, we performed differential expression analyses and screened out the intersecting DEGs. Thus, we defined four genes that were overexpressed from ductal cells to transitional ductal cells and PASC cells: LGALS1, NPM1, RACK1, and PERP (Figure 5C and D).

### The pseudotime trajectory of ductal and PASC cells

**Figure 6:**
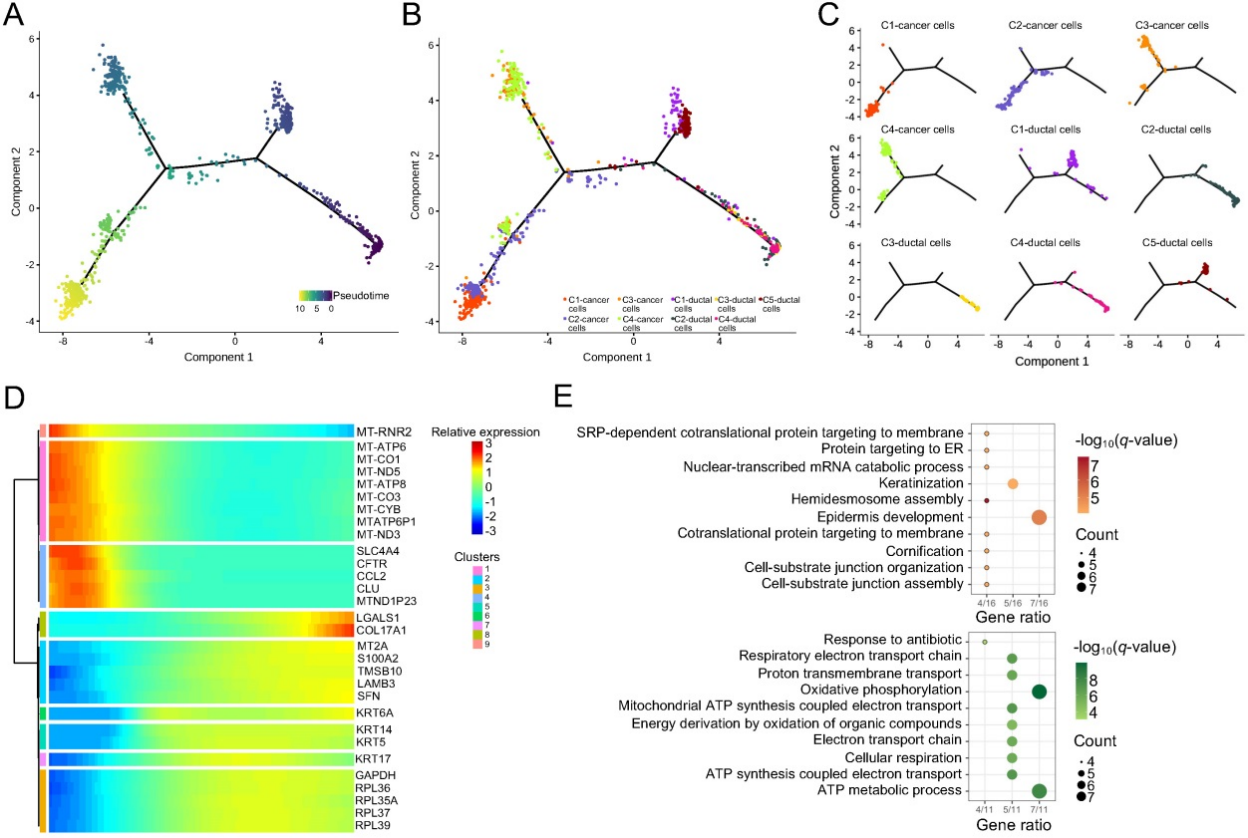
** Pseudotime analyses of ductal and PASC cells.** (**A**) Trajectory charts showing the differential stages of ductal and cancer cells (dark and light colors indicate early and late stages, respectively). (**B**) and (**C**) Four cancer cell clusters and five duct-cell clusters matching the trajectory chart. (**D**) Differential gene expression panel from ductal to cancer cells. (**E**) The biological process of upregulated (up) and downregulated (down) gene sets in PASC progression.

Pseudotime analyses were conducted based on the scRNA-seq data of five ductal and four carcinoma cell clusters. This analysis revealed three evolutionary dynamic states with distinct genotypes in these cells (Figure 6A). In the initial state, the ductal cells and PASC cells expressed pancreatic feature genes, such as SPINK1 and REG3A (Table S4). SPINK1 a trypsin inhibitor suppresses the activation of zymogens via trypsin within the pancreatic duct. REG3A encodes a pancreatic secretory protein associated with pancreatic inflammation and antimicrobial activity. C-1 and C-5 ductal cells differentiated into the first cell state, expressing SPRR3 and showing keratinization characteristics (Table S5). C-3 and C-4 cancer cells exhibited the gene pattern of the second cell state however, we could not identify the specific functional genes within these cells (Table S6). C-1 and C-2 cancer cells finally differentiated into the third cells state, which expressed SFRP5, a Wnt-signaling regulator (Table S7). Overall, the formation of cancer cells appeared after the evolution of normal ductal cells. Four cancer-cell clusters evolved along two differentiation trajectories whereas ductal cells followed one evolutionary state (Figure 6B and C). During pseudotime, the significantly altered genes with similar expression models were clustered into nine subsets (Figure 6D). We further performed biological process analyses for these genes. The results indicated that the downregulated gene set was associated with “ATP metabolic process” (Figure 6E) 28. Conversely, the upregulated gene set was mainly enriched in “epidermal development” and “keratinization” (Figure 6E), which was probably due to the adenosquamous properties of the tumor sample used in this study.

### Features of the five PASC cell subgroups

**Figure 7:**
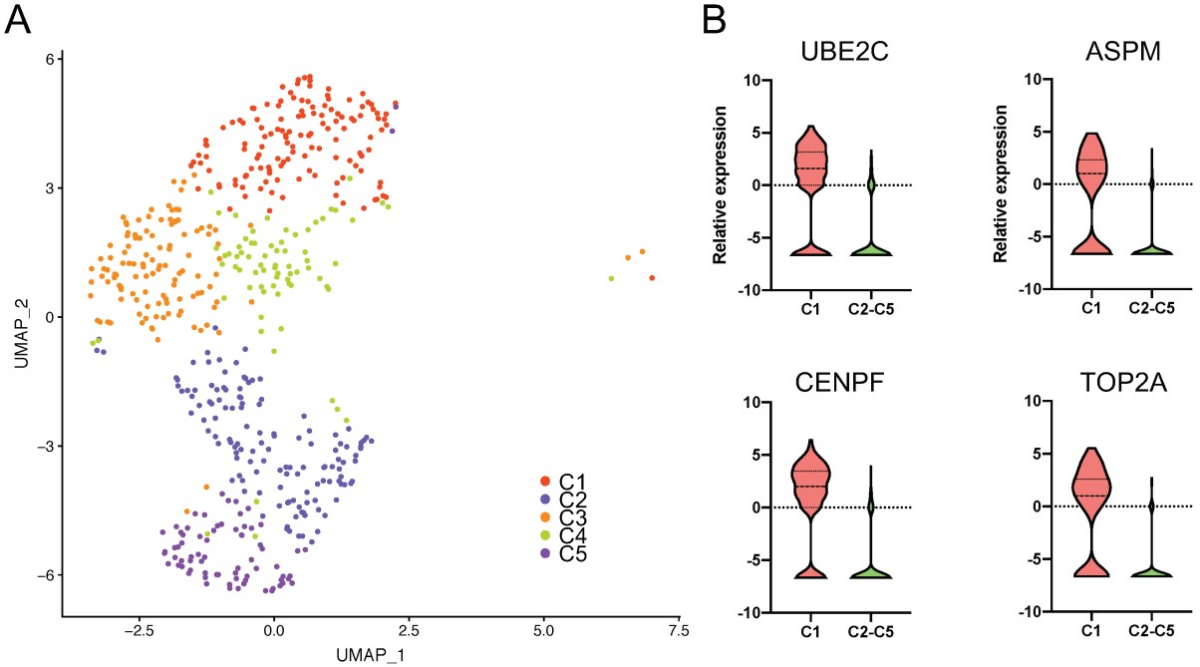
** The marker genes of stem-like cancer cells within PASC.** (**A**) tSNE plot showing five distinct cell types from PASC. (**B**) UBE2C, ASPM, CENPF, and TOP2A were highly expressed in stem-like cancer cells (C1) compared with the other clusters.

We identified 559 cancer cells according to epithelial and cancer markers including KRT19, KRT7, EPCAM, SOX9, and KRT23. Then, we grouped the cells into five clusters (C1-C5) based on specific gene patterns (Figure 7A).

Notably, we observed that UBE2C, ASPM, CENPF, and TOP2A were significantly overexpressed in C1 compared to the other clusters. This cluster exhibited distinct karyokinetic and proliferation activity (Figure 7B and Table S8). For example, UBE2C maintains cell stemness in hepatocellular carcinoma 29. The stem cell marker, ASPM, promotes cell proliferation in prostate cancer 30 through the Wnt signaling pathway 28, and CENPF is involved in the formation of the centromere-kinetochore complex and was correlated with poor prognosis in the PDAC cohort of TCGA (Figure S4A). TOP2A was upregulated in tumor tissues 31, correlated with the poor survival of PC patients (Figure S4B), and induced PC cell progression 32. Furthermore, NUSAP1 and TPX2, as C1 marker genes, contribute to spindle organization. The high TPX2 expression was associated with oncogenic KRAS mutations, and TPX2 inhibition suppresses PC cell growth 33. Additionally, SMC4 promotes glioma cell proliferation through TGF-β 34. Since C1 specifically contains stem cell and proliferative genes, we regarded this cluster as stem-like cancer cells, which suggests that drugs targeting these marker genes inhibit PASC proliferation.

We also observed that C2 expressed several transcription factors, including MXD1, ELF3, and LMO7 (Table S9). MXD1 negatively regulates gastric cancer cell migration, invasion, and metastasis, and serves as a c-Myc antagonist 35. ELF3 stability is associated with PDAC progression via endogenous ERK1/2 phosphorylation stimulation 36. Additionally, LMO7 was upregulated in papillary thyroid carcinoma and is a regulatory factor that promotes cell growth by fusing with BRAF 37.

We discovered the overexpression of laminins (LAMA3, LAMC2, and LAMB3), MMP1, and SERPINE1 in C3 (Table S10), suggesting that this cell type participates in microenvironmental reconstruction. For example, MMP1 is involved in extracellular matrix breakdown and is usually upregulated during pancreatic tumorigenesis 38. Similarly, LAMA3 was increased in tumor tissues and correlated with the poor prognosis of PDAC patients 39, while SERPINE1 promoted PC cell proliferation and invasion 40.

Unexpectedly, C4, like dormancy, expressed no specific molecular markers. Indeed, we only observed the downregulation of several tumor-suppressive genes including RHCG 41 and DUSP1 42 and oncogenes including EMP1 43 and S100P 44 (Table S11). Since the pathological tumor tissue was adenosquamous carcinoma, we observed several keratins, including KRT1, KRT6B, KRTDAP, and KRT10, in C5 (Table S12).

### Overexpression of EREG, FCGR2A, CCL4L2, and CTSC in myeloid cells from the normal pancreas to PASC

**Figure 8:**
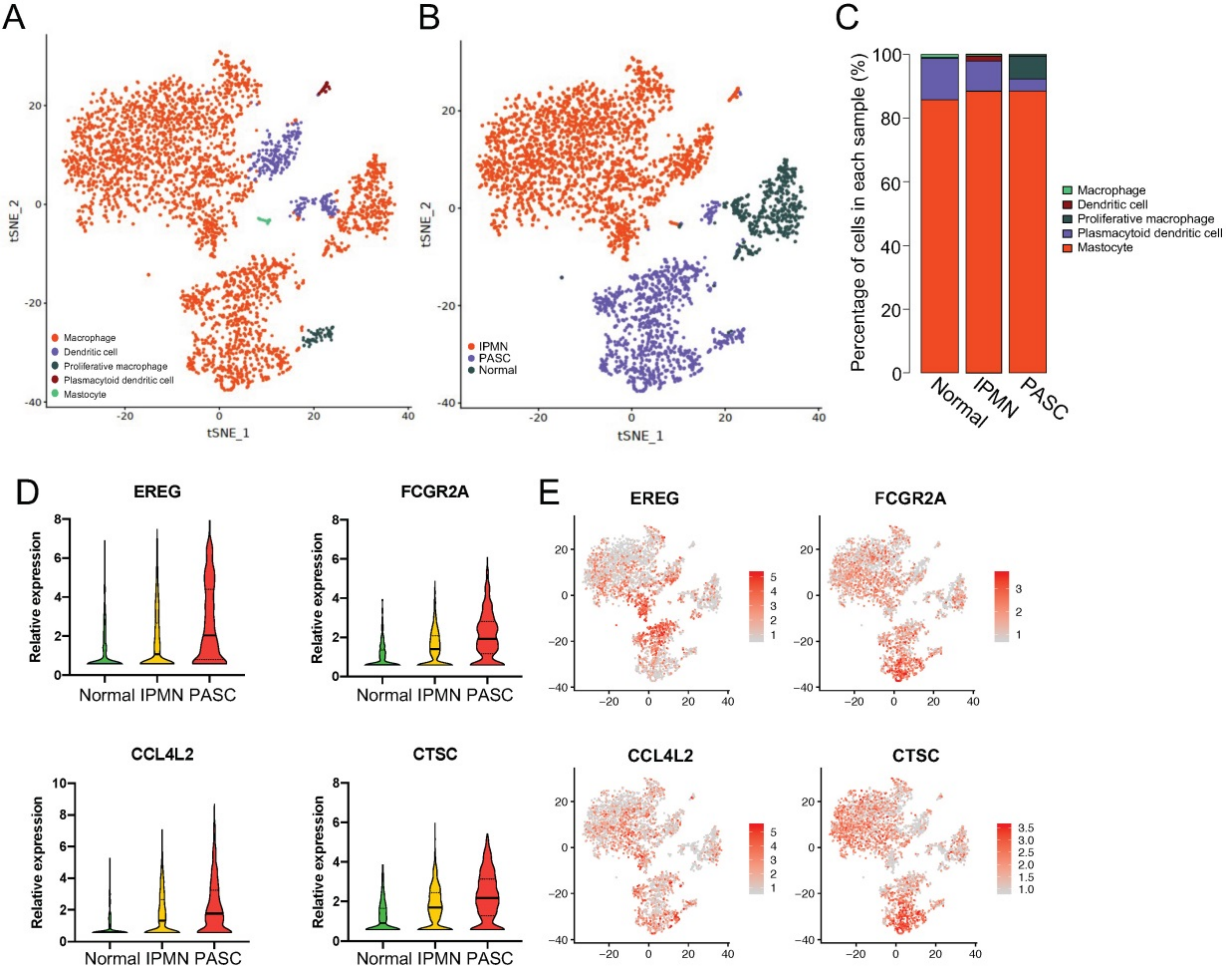
** The upregulated genes of myeloid cells across the normal pancreas, IPMN, and PASC.** (**A**) tSNE plots identifying five myeloid cell subgroups. (**B**) and (**C**) The distribution and percentage of each cell subset within myeloid cells. (**D**) and (**E**) The expression of EREG, FCGR2A, CCL4L2, and CTSC increases from the normal pancreas to PASC.

Since most stromal cells were myeloid cells, we investigated the gene variations in this cell type to predict its effect on PASC development. Myeloid cells were divided into five subgroups: macrophages (88.2%), dendritic cells (8.3%), proliferative macrophages (2.0%), mastocytes (0.5), and plasmacytoid dendritic cells (1.0%; Figure 8A-C).

For macrophages, we identified four genes that were upregulated from the healthy pancreas to IPMN and PASC (Figure 8D and E): EREG, which encodes an epidermal growth factor receptor ligand that promotes carcinogenesis 45, 46; FCGR2A, which encodes a macrophage surface receptor that is involved in phagocytosis and clearance of immune complexes and exhibits polymorphisms associated with chemotherapy efficacy in KRAS-mutated tumors 47; CCL4L2, which encodes cytokine proteins and functions in inflammatory and immunoregulatory processes; and CTSC, which encodes a member of the peptidase C1 family that activates serine proteinases. In hepatocellular carcinoma cells, CTSC accelerates proliferation and metastasis by activating the TNF-α/p38 pathway 48. CTSC inhibition was also reported to positively regulate autophagy 49. Thus, we discovered aberrant changes in myeloid cells, suggesting that blocking myeloid cell progression inhibits PASC tumorigenesis.

### Cancer-associated fibroblasts (CAFs) restore an immunosuppressive tumor microenvironment

**Figure 9:**
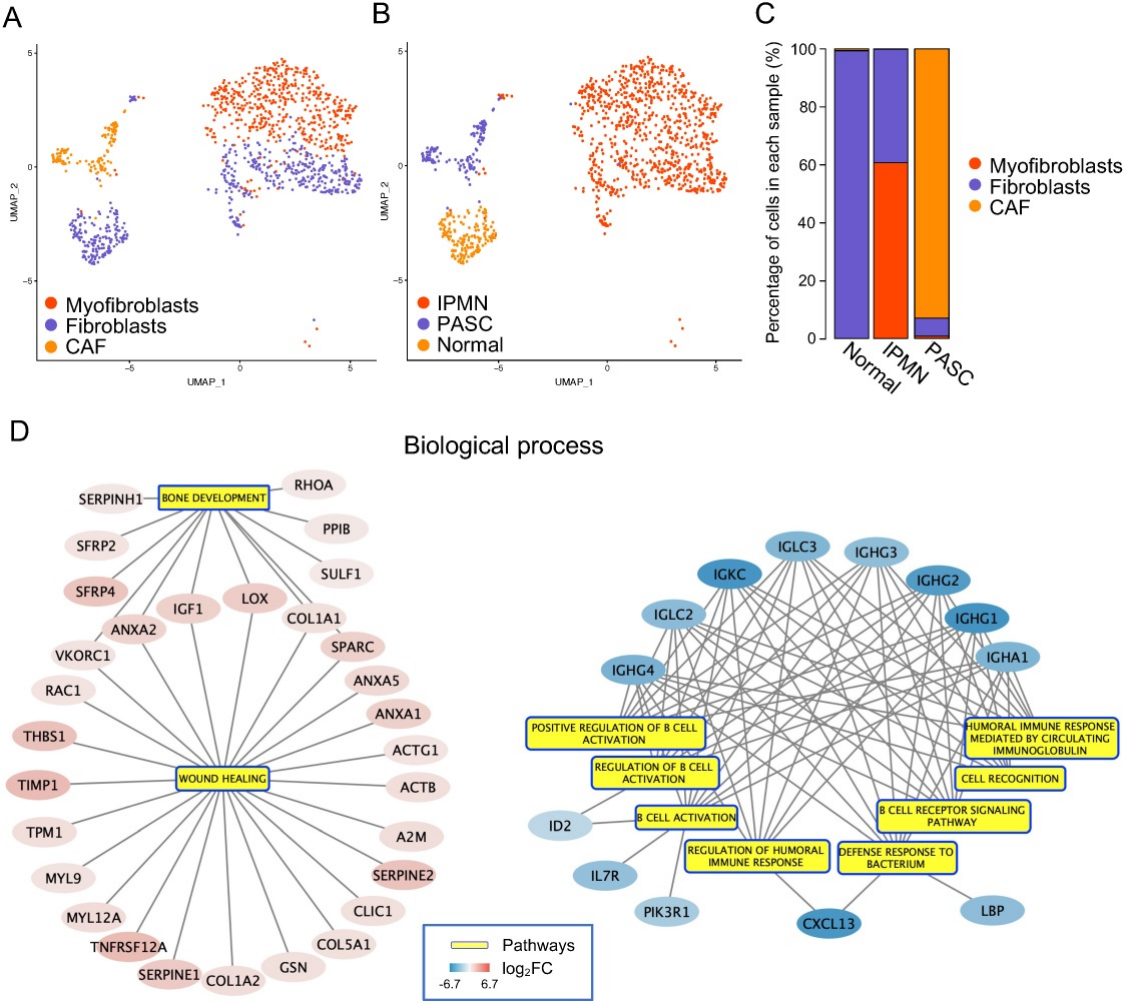
** Functions of cancer-associated fibroblasts in PASC.** (**A**) tSNE plot showing fibroblasts, myofibroblasts, and CAFs. (**B**) and (**C**) The distribution and percentage of each fibroblast subgroup. (**D**) The biological process of DEGs between CAFs and fibroblasts (left: upregulated gene set; right: downregulated gene set).

To delineate fibroblast heterogeneity within the tumor microenvironment, we subdivided 1,135 cells into fibroblasts, myofibroblasts, and CAFs (Figure 9A-C). The gene markers for each cell type were selected based on the results of previous studies 50, 51, and are summarized in Table S13. We performed differential expression analysis between fibroblasts and CAFs to uncover the aberrant functions within CAFs. The GSEA results showed that in biological processes, enriched DEGs were negatively associated with the “B cell activation and immune response” and positively associated with “bone development and wound healing” (Figure 9D). For molecular function, this gene set was involved in "structural constituent of the cytoskeleton," "calcium ion binding," and "extracellular matrix structure constituent," which participate in the reconstitution of extra- and intracellular microenvironments (Figure S5). In contrast, the DEGs were negatively correlated with "antigen-binding," which enhances the association between CAFs and the immune response (Figure S5). In cellular components, 25 DEGs were related to an accelerated “actin cytoskeleton,” which plays an essential role in cell migration (Figure S6). Consequently, we revealed that CAFs may support tumor progression by rebuilding the immunosuppressive microenvironment.

### The copy number variation landscape between ductal and PASC cells

**Figure 10:**
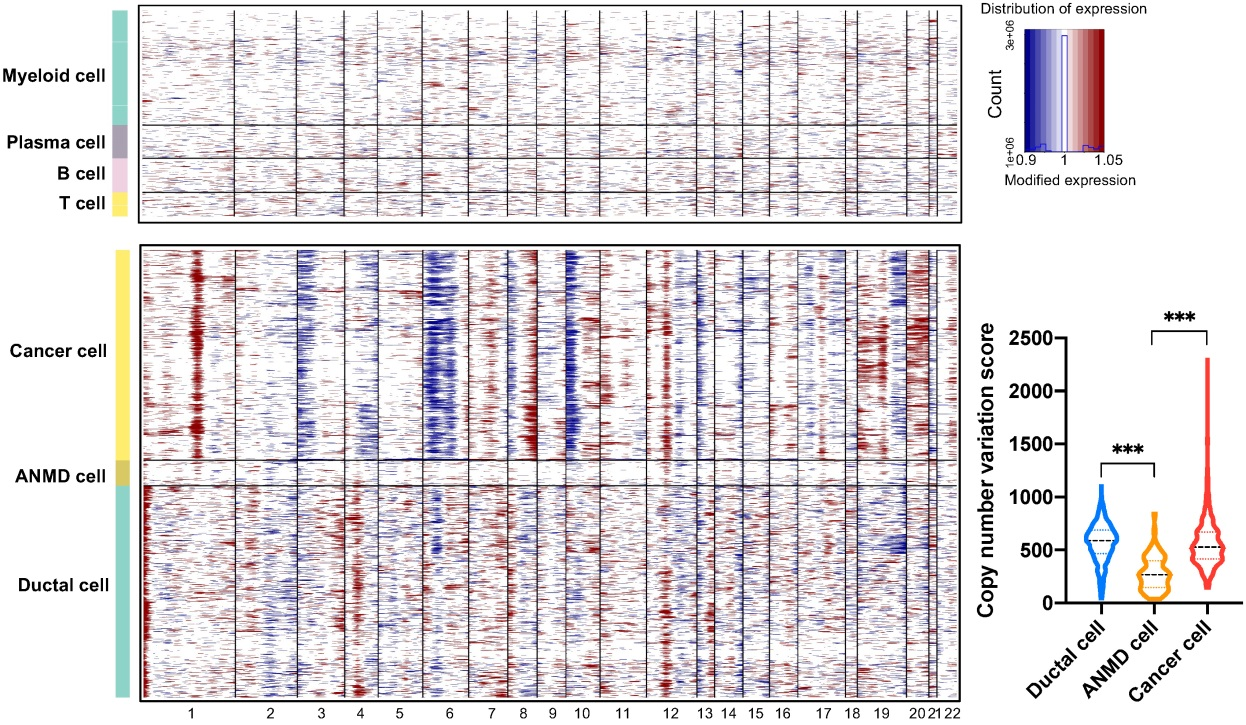
** Copy number variation profiles of ductal, adjacent nonmalignant, and PASC cells.** Large-scale CNVs of reference cells including myeloid, plasma, B, and T cells (top); PASC, adjacent nonmalignant ductal (ANMD), and ductal cells (bottom). The red and blue colors represent high and low CNV scores, respectively. The CNV levels of PASC and ductal cells exceed that of ANMD cells (low right). ***P < 0.001. Data are presented as mean ± SD.

To investigate large-scale chromosomal changes among ductal (563 cells), adjacent nonmalignant (68 cells), and PASC cells (559 cells), we performed CNV analyses based on our scRNA-seq data. The results demonstrated that compared with reference cells (myeloid, B, and T cells), DNA insertions in cancer cells mainly occurred in chromosomes 1, 8, 19, and 20, while deletions were present in chromosomes 3, 6, and 10 in (Figure 10). Notably, the total CNV score significantly increased in ductal and cancer cells compared with that in ANMD cells. Although the CNV score was comparable between ductal and cancer cells, the CNV features between these two cell types differed (Figure 10).

### Activation of EGFR-associated ligand-receptor pairs among fibroblast, myeloid cells, and PASC cells

**Figure 11:**
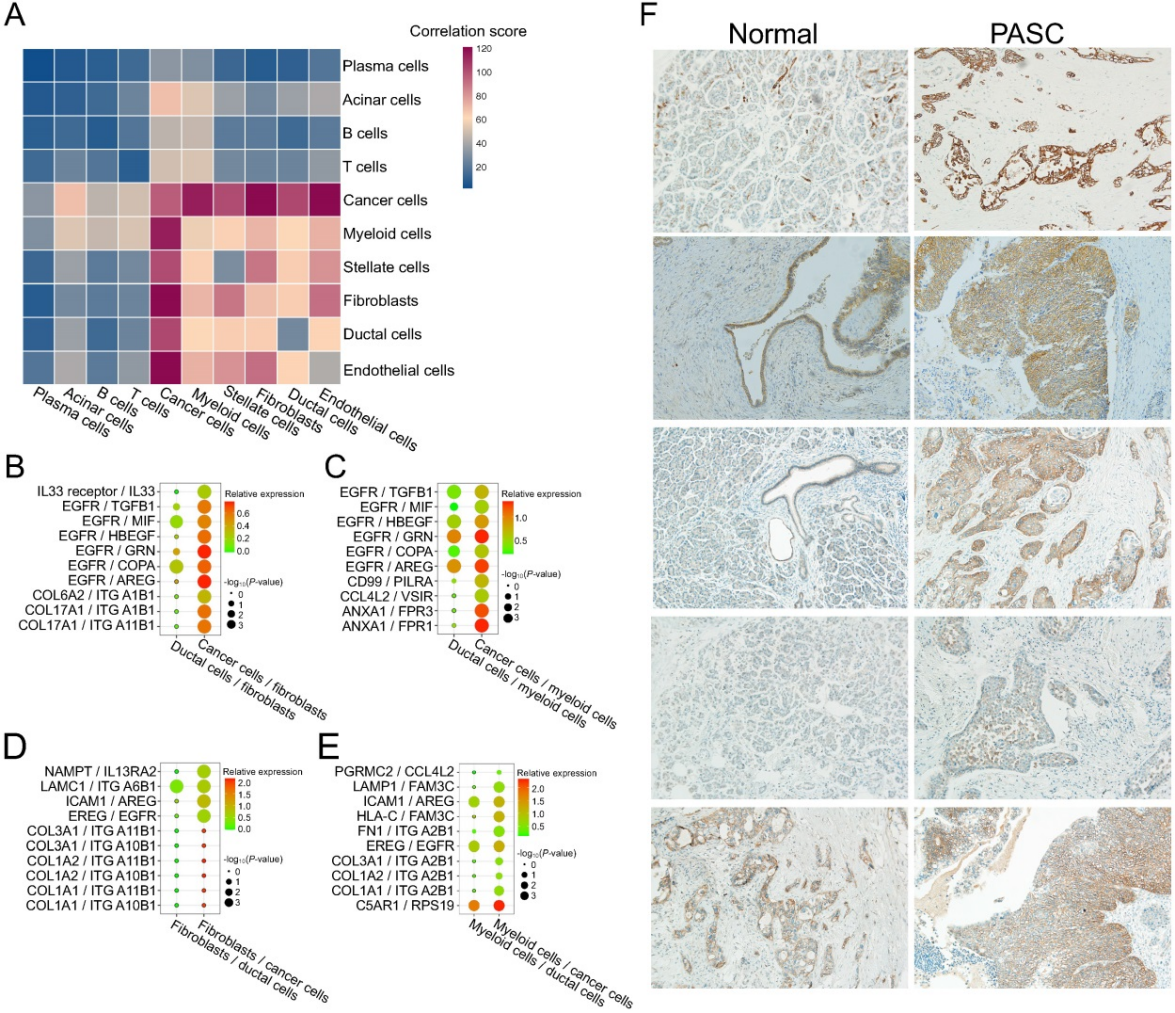
** Cell-cell communication between stromal and PASC cells.** (**A**) Heatmap exhibiting cell-cell communication scores. Dark red and blue colors represent high and low levels of interactional intensity, respectively. Activated intercellular signaling between fibroblasts and cancer cells (**B**), myeloid cells and cancer cells (**C**), fibroblasts and cancer cells (**D**), and myeloid cells and cancer cells (**E**) compared with ductal cells. (**F**) EGFR immunohistochemical staining of five paired PASC tissues. (200×)

To examine the intercellular interactions during PASC progression, we performed cell-cell communication analyses based on scRNA-seq data and the CellPhoneDB database. The significance and expression level of the ligand-receptor of fibroblasts and myeloid cells to ductal cells are presented and visualized in Figure S7. Similarly, we showed the expression level of the ligand-receptor of fibroblasts and myeloid cells to cancer cells (Figure S8). Thus, we selected the significantly changed ligand-receptor pairs between cancer and stromal cells from this cell-cell interaction network. The results revealed that PASC cells communicate more closely with myeloid cells and fibroblasts than other cell types (Figure 11A). We further screened the activated ligand-receptor pairs between ductal cells and these two stroma-cell types and defined several cancer-associated pairs, such as EGFR/TGFB1, ANXA1/FPR3, EREG/EGFR, and ICAM1/AREG (Figure 11B-E). Notably, EGFR and its receptors, including TGFB1, MIF, HBEGF, GRN, COPA, and AREG, were upregulated in cancer cells and fibroblasts. Consequently, we identified an EGFR-associated feedback loop that promotes PASC progression from ductal cells to cancer cells. EGFR in cancer cells is overexpressed, thereby activating fibroblasts and myeloid cells via several molecules such as AREG. AREG reacts with cancer cells and results in the activation of EGFR in pancreatic cancer 52. This crosstalk between fibroblasts, myeloid cells, and cancer cells may provide a potential therapeutic target for PASC. Besides, several cancer-associated ligand-receptor pairs, such as NAMPT/IL13RA2, were also overexpressed in the interactions between fibroblasts and cancer cells (Figure 11D) 53. Similarly, myeloid cells communicate with cancer cells via C5AR1/RPS19 (Figure 11E). Finally, immunohistochemistry results showed that EGFR stains stronger in PASC tissues than in adjacent normal tissues (Figure 11F). Thus, our findings supported that anti-EGFR could be a potential drug therapy for PASC.

## Discussion

PASC is a rare but highly aggressive type of pancreatic cancer, containing abundant desmoplastic stroma and a small fraction of tumor cells. Therefore, assessing the transcriptome patterns of PASC cells in a bulk tumor remains a challenge. Recently, scRNA-seq has emerged as a new method to illustrate the cellular heterogeneity within pancreatic cancer 7. In this study, using scRNA-seq, we identified S100A2 as a novel biomarker for PASC cells; discovered cells of a stem-like (C1), transcription factor-activated (C2), and microenvironmental reconstruction-associated (C3) types within PASC cells; defined EREG, FCGR2A, CCL4L2, and CTSC as feature genes of myeloid cells ranging from the normal pancreas to IPMN and PASC; revealed that CAFs may be associated with the reconstruction of immunosuppressive microenvironment; depicted the CNV of PASC cells; and discovered EGFR activation in cell-cell communication between PASC and stromal cells. Finally, these findings explain the heterogeneity and underlying cancer-associated regulators in PASC.

Recently, several studies have deciphered the heterogeneity of cancer and stromal cells of PC to identify the potential biomarkers and therapeutic targets for treating this lethal cancer, using scRNA-seq 7, 51, 54. However, transcriptome differences between normal ductal and PASC cells are generally not examined due to the inaccessibility of a healthy pancreas and the rarity of PASC. Additionally, owing to tumor heterogeneity, the effects of the tumor microenvironment on carcinogenesis are poorly understood. To overcome these problems, we harvested pancreatic cells from a healthy donor and dissected the main pancreatic duct to obtain more living ductal cells. Hence, to our knowledge, this is the first study to compare the differences between PASC and normal pancreatic ductal cells using scRNA-seq.

Clinically, early PASC detection is difficult because no specific and sensitive molecular markers are present in tumorigenesis. At the single-cell level, we defined S100A2 as a potential biomarker for cancer cells in PASC. Besides, we revealed that S100A2 was associated with undesirable clinical features in PC. According to a recent study, S100A2 is upregulated in bulk tissues and could be an independent prognostic factor for patients with PDAC 55. Therefore, our findings indicate that anti-S100A2 therapy may be a novel treatment strategy for PASC.

GSEA analyses also revealed several recognized cancer-driving activation pathways in PASC cells, such as VEGFA/VEGFR2 56, 57, glycolysis/gluconeogenesis 58, and oxidative phosphorylation 59. In contrast, two genes were involved in EMT suppression; GDF15 exhibits antitumor effects 60, and PROX1, which is involved in DNA binding transcriptional activity, protects pancreatic cells from acute tissue damage and early neoplastic transformation 61. Besides, we observed distinct CNV patterns in ductal cells compared to reference cells, suggesting the intrinsic heterogeneity of diverse cell types.

Meanwhile, the expression of four genes increased from ductal cells to transitional ductal and malignant cells. LGALS1, a member of the beta-galactoside-binding protein family, is upregulated in PDAC stroma, promoting microenvironmental vascularization 62. Mechanistically, LGALS1 may activate SDF-1 through NF-κB, thereby increasing pancreatic stellate cell migration and invasion 63. NPM1, which is associated with tumor metastasis, is highly expressed in lung adenocarcinoma 64. As a ribosomal protein, RACK1 promotes tumor progression in breast cancer and oral squamous cell carcinoma 65, 66. However, as a molecule downstream of METTL14, PERP can inhibit PC cell proliferation and migration 67. Thus, these four genes may be driving regulators and prognostic biomarkers for PASC development.

Pseudotime trajectory analysis revealed that several genes may change along with the differentiation process from ductal to PASC cells. For example, CFTR —which encodes a member of the ATP-binding cassette transporter superfamily that functions as a chloride channel and controls ion and water secretion in epithelial tissues — decreased from ductal to cancer cells. Mutations of CFTR cause cystic fibrosis — a risk factor for PC 28. Besides, the biological process analysis indicated that the highly expressed gene set was associated with cellular substrate organization (Figure 6E), a crucial feature of PC progression.

Another valuable observation from our scRNA-seq data is the evaluation of cell-cell communication based on ligand-receptor expressions. For example, the level of the EGFR/TGFB1 pair was overexpressed from ductal cell-fibroblast to cancer cell-fibroblast communication (Figure 11B). EGFR could alter TGF-β function from antitumorigenic to protumorigenic in breast cancer 68, while TGFB1 increased fibrogenic gene expression and myofibroblast differentiation 69. Similarly, the expression of the ANXA1/FPR1 or FPR3 pair was upregulated from ductal cell-myeloid cell to cancer cell-myeloid cell communication (Figure 11C). ANXA1 is an anti-inflammatory molecule in PC 70. FPR1 and FPR3 are macrophage gene markers, participating in neutrophil recruitment 71 and tumor-associated inflammation 72. Thus, our findings highlighted the signaling pathways between cancer and stromal cells.

We also revealed the crosstalk between fibroblasts and cancer cells via ICAM1/AREG and collagen/integrin. As a molecule downstream of IL-35, ICAM1 facilitates endothelial adhesion and transendothelial migration, which accelerates PC metastasis 73. As an epidermal growth factor, AREG significantly influences EMT upregulation in PC via the EGFR signaling pathway 74. The expression of COL1A1, COL1A2, and COL3A1, members of the collagen family, is increased in lung adenocarcinomas and is associated with its poor prognosis. Since the activation of integrin signaling is correlated with cancer metastasis 75, our findings indicate that the ligand-receptor interaction between fibroblasts and cancer cells could contribute to PASC migration.

Myeloid cell-cancer cell communication analyses revealed activation of C5AR1/RPS19 and HLA-C/FAM3C pairs. RPS19 is upregulated in breast and ovarian cancer cells and promotes the presence of regulatory T cells, reducing CD8 T cell infiltration 76. Similarly, C5AR1 recruits myeloid-derived suppressor cells into inflamed colorectum to impair CD8 T cells 77. Thus, activating C5AR1/RPS19 may induce immunosuppression during PC progression. As a member of the family with sequence similarity 3, FAM3C encodes a secreted protein that is upregulated in pancreatic cancer cells 78, 79. Consequently, the overexpression of intercellular signaling among fibroblasts, myeloid cells, and cancer cells uncover novel biological insights into abnormal cell-cell communications in PASC.

This study has some limitations; for example, due to ischemia and necrosis, most epithelial cells in the IPMN group did not undergo quality control. Therefore, we could not compare the gene patterns of ductal cells among all three sample tissues. Besides, we did not explore T, B, and plasma cells since they were only present in IPMN, and a continuous change in the gene pattern was not observed. In addition, each type of tissue sample comes from a single patient, which may affect the credibility of this study. Thus, a larger number of PASC samples and their respective stages are needed to thoroughly understand the progression of PASC.

## Conclusion

We examined the ductal and stromal cell variations during PASC progression from a single-cell perspective. We identified novel molecular markers for cancer cells, defined the underlying cancer-associated signaling pathways, and deciphered cell-cell communication during PASC tumorigenesis. Collectively, these results provide valuable information to understand the critical biological processes underlying PASC and demonstrate potential therapeutic targets for PASC.

## Supplementary Material

Supplementary figures and tables.

## Abbreviations

PASC: pancreatic adenosquamous carcinoma
PDAC: pancreatic ductal adenocarcinoma
PC: pancreatic cancer
scRNA-seq: single-cell RNA sequencing
IPMN: intraductal papillary mucinous neoplasm
UMI: unique molecular identifier
tSNE: t-distribution stochastic neighbor embedding
DEGs: differentially expressed genes
GSEA: gene set enrichment analysis
ANMD cells: adjacent nonmalignant ductal cells
CNV: copy number variation
TCGA: The Cancer Genome Atlas
EMT: epithelial-mesenchymal transition
CAF: cancer-associated fibroblast

## References

[1] Kleeff J, Korc M, Apte M (2016). Pancreatic cancer. Nat Rev Dis Primers.

[2] Hester CA, Augustine MM, Choti MA (2018). Comparative outcomes of adenosquamous carcinoma of the pancreas: An analysis of the National Cancer Database. J Surg Oncol.

[3] Boecker J, Feyerabend B, Tiemann K (2020). Adenosquamous Carcinoma of the Pancreas Comprise a Heterogeneous Group of Tumors With the Worst Outcome: A Clinicopathological Analysis of 25 Cases Identified in 562 Pancreatic Carcinomas Resected With Curative Intent. Pancreas.

[4] Fang Y, Su Z, Xie J (2017). Genomic signatures of pancreatic adenosquamous carcinoma (PASC). J Pathol.

[5] Brody JR, Costantino CL, Potoczek M (2009). Adenosquamous carcinoma of the pancreas harbors KRAS2, DPC4 and TP53 molecular alterations similar to pancreatic ductal adenocarcinoma. Mod Pathol.

[6] Bernard V, Semaan A, Huang J (2019). Single-Cell Transcriptomics of Pancreatic Cancer Precursors Demonstrates Epithelial and Microenvironmental Heterogeneity as an Early Event in Neoplastic Progression. Clin Cancer Res.

[7] Peng J, Sun BF, Chen CY (2019). Author Correction: Single-cell RNA-seq highlights intra-tumoral heterogeneity and malignant progression in pancreatic ductal adenocarcinoma. Cell Res.

[8] Ding L, Su Y, Fassl A (2019). Perturbed myoepithelial cell differentiation in BRCA mutation carriers and in ductal carcinoma *in situ*. Nat Commun.

[9] Liao Y, Smyth GK, Shi W (2014). featureCounts: an efficient general purpose program for assigning sequence reads to genomic features. Bioinformatics.

[10] Tsuyuzaki K, Sato H, Sato K (2020). Benchmarking principal component analysis for large-scale single-cell RNA-sequencing. Genome Biol.

[11] Kobak D, Berens P (2019). The art of using t-SNE for single-cell transcriptomics. Nat Commun.

[12] Efremova M, Vento-Tormo M, Teichmann SA (2020). CellPhoneDB: inferring cell-cell communication from combined expression of multi-subunit ligand-receptor complexes. Nat Protoc.

[13] He W, Yuan T, Maedler K (2019). Macrophage-associated pro-inflammatory state in human islets from obese individuals. Nutr Diabetes.

[14] Sánchez-Espiridión B, Martin-Moreno AM, Montalbán C (2012). Immunohistochemical markers for tumor associated macrophages and survival in advanced classical Hodgkin's lymphoma. Haematologica.

[15] Edlund K, Madjar K, Mattsson JSM (2019). Prognostic Impact of Tumor Cell Programmed Death Ligand 1 Expression and Immune Cell Infiltration in NSCLC. J Thorac Oncol.

[16] O'Connor D, Clutterbuck EA, Thompson AJ (2017). High-dimensional assessment of B-cell responses to quadrivalent meningococcal conjugate and plain polysaccharide vaccine. Genome Med.

[17] Oostindie SC, van der Horst HJ, Lindorfer MA (2019). CD20 and CD37 antibodies synergize to activate complement by Fc-mediated clustering. Haematologica.

[18] Binder C, Cvetkovski F, Sellberg F (2020). CD2 Immunobiology. Front Immunol.

[19] Krämer M, Plum PS, Velazquez Camacho O (2020). Cell type-specific transcriptomics of esophageal adenocarcinoma as a scalable alternative for single cell transcriptomics. Mol Oncol.

[20] Aue A, Hinze C, Walentin K (2015). A Grainyhead-Like 2/Ovo-Like 2 Pathway Regulates Renal Epithelial Barrier Function and Lumen Expansion. J Am Soc Nephrol.

[21] Stoy C, Sundaram A, Rios Garcia M (2015). Transcriptional co-factor Transducin beta-like (TBL) 1 acts as a checkpoint in pancreatic cancer malignancy. EMBO Mol Med.

[22] Bridel C, Koel-Simmelink MJA, Peferoen L (2018). Brain endothelial cell expression of SPARCL-1 is specific to chronic multiple sclerosis lesions and is regulated by inflammatory mediators *in vitro*. Neuropathol Appl Neurobiol.

[23] Roudnicky F, Zhang JD, Kim BK (2020). Inducers of the endothelial cell barrier identified through chemogenomic screening in genome-edited hPSC-endothelial cells. Proc Natl Acad Sci U S A.

[24] Chiu IJ, Hsu YH, Chang JS (2020). Lactotransferrin Downregulation Drives the Metastatic Progression in Clear Cell Renal Cell Carcinoma. Cancers (Basel).

[25] Otomo R, Otsubo C, Matsushima-Hibiya Y (2014). TSPAN12 is a critical factor for cancer-fibroblast cell contact-mediated cancer invasion. Proc Natl Acad Sci U S A.

[26] Takehara M, Sato Y, Kimura T (2020). Cancer-associated adipocytes promote pancreatic cancer progression through SAA1 expression. Cancer Sci.

[27] Huang M, Wu R, Chen L (2019). S100A9 Regulates MDSCs-Mediated Immune Suppression via the RAGE and TLR4 Signaling Pathways in Colorectal Carcinoma. Front Immunol.

[28] McWilliams RR, Petersen GM, Rabe KG (2010). Cystic fibrosis transmembrane conductance regulator (CFTR) gene mutations and risk for pancreatic adenocarcinoma. Cancer.

[29] Song Y, Kim S, Lee H (2020). Chromenopyrimidinone Controls Stemness and Malignancy by suppressing CD133 Expression in Hepatocellular Carcinoma. Cancers (Basel).

[30] Pai VC, Hsu CC, Chan TS (2019). ASPM promotes prostate cancer stemness and progression by augmenting Wnt-Dvl-3-β-catenin signaling. Oncogene.

[31] Heestand GM, Schwaederle M, Gatalica Z (2017). Topoisomerase expression and amplification in solid tumours: Analysis of 24,262 patients. Eur J Cancer.

[32] Pei YF, Yin XM, Liu XQ (2018). TOP2A induces malignant character of pancreatic cancer through activating β-catenin signaling pathway. Biochim Biophys Acta Mol Basis Dis.

[33] Gomes-Filho SM, Dos Santos EO, Bertoldi ERM (2020). Aurora A kinase and its activator TPX2 are potential therapeutic targets in KRAS-induced pancreatic cancer. Cell Oncol (Dordr).

[34] Jiang L, Zhou J, Zhong D (2017). Overexpression of SMC4 activates TGFβ/Smad signaling and promotes aggressive phenotype in glioma cells. Oncogenesis.

[35] Wu Q, Yang Z, An Y (2014). MiR-19a/b modulate the metastasis of gastric cancer cells by targeting the tumour suppressor MXD1. Cell Death Dis.

[36] Feng Y, Gao L, Cui G (2020). LncRNA NEAT1 facilitates pancreatic cancer growth and metastasis through stabilizing ELF3 mRNA. Am J Cancer Res.

[37] He H, Li W, Yan P (2018). Identification of a Recurrent LMO7-BRAF Fusion in Papillary Thyroid Carcinoma. Thyroid.

[38] Huang C, Li Y, Guo Y (2018). MMP1/PAR1/SP/NK1R paracrine loop modulates early perineural invasion of pancreatic cancer cells. Theranostics.

[39] Pan Z, Li L, Fang Q (2018). Analysis of dynamic molecular networks for pancreatic ductal adenocarcinoma progression. Cancer Cell Int.

[40] Botla SK, Savant S, Jandaghi P (2016). Early Epigenetic Downregulation of microRNA-192 Expression Promotes Pancreatic Cancer Progression. Cancer Res.

[41] Ming XY, Zhang X, Cao TT (2018). RHCG Suppresses Tumorigenicity and Metastasis in Esophageal Squamous Cell Carcinoma via Inhibiting NF-κB Signaling and MMP1 Expression. Theranostics.

[42] Chen Z, Chen Q, Cheng Z (2020). Long non-coding RNA CASC9 promotes gefitinib resistance in NSCLC by epigenetic repression of DUSP1. Cell Death Dis.

[43] Ahmat Amin MKB, Shimizu A, Zankov DP (2018). Epithelial membrane protein 1 promotes tumor metastasis by enhancing cell migration via copine-III and Rac1. Oncogene.

[44] Dakhel S, Padilla L, Adan J (2014). S100P antibody-mediated therapy as a new promising strategy for the treatment of pancreatic cancer. Oncogenesis.

[45] Liu S, Wang Y, Han Y (2020). EREG-driven oncogenesis of Head and Neck Squamous Cell Carcinoma exhibits higher sensitivity to Erlotinib therapy. Theranostics.

[46] Wang Y, Jing Y, Ding L (2019). Epiregulin reprograms cancer-associated fibroblasts and facilitates oral squamous cell carcinoma invasion via JAK2-STAT3 pathway. J Exp Clin Cancer Res.

[47] Kjersem JB, Skovlund E, Ikdahl T (2014). FCGR2A and FCGR3A polymorphisms and clinical outcome in metastatic colorectal cancer patients treated with first-line 5-fluorouracil/folinic acid and oxaliplatin +/- cetuximab. BMC Cancer.

[48] Zhang GP, Yue X, Li SQ (2020). Cathepsin C Interacts with TNF-α/p38 MAPK Signaling Pathway to Promote Proliferation and Metastasis in Hepatocellular Carcinoma. Cancer Res Treat.

[49] Khaket TP, Singh MP, Khan I (2018). Targeting of cathepsin C induces autophagic dysregulation that directs ER stress mediated cellular cytotoxicity in colorectal cancer cells. Cell Signal.

[50] Wu SZ, Roden DL, Wang C (2020). Stromal cell diversity associated with immune evasion in human triple-negative breast cancer. Embo j.

[51] Elyada E, Bolisetty M, Laise P (2019). Cross-Species Single-Cell Analysis of Pancreatic Ductal Adenocarcinoma Reveals Antigen-Presenting Cancer-Associated Fibroblasts. Cancer Discov.

[52] Nagathihalli NS, Beesetty Y, Lee W (2014). Novel mechanistic insights into ectodomain shedding of EGFR Ligands Amphiregulin and TGF-α: impact on gastrointestinal cancers driven by secondary bile acids. Cancer Res.

[53] Chong ST, Tan KM, Kok CYL (2019). IL13RA2 Is Differentially Regulated in Papillary Thyroid Carcinoma vs Follicular Thyroid Carcinoma. J Clin Endocrinol Metab.

[54] Kuboki Y, Fischer CG, Beleva Guthrie V (2019). Single-cell sequencing defines genetic heterogeneity in pancreatic cancer precursor lesions. J Pathol.

[55] Dreyer SB, Pinese M, Jamieson NB (2020). Precision Oncology in Surgery: Patient Selection for Operable Pancreatic Cancer. Ann Surg.

[56] Momeny M, Alishahi Z, Eyvani H (2020). Anti-tumor activity of cediranib, a pan-vascular endothelial growth factor receptor inhibitor, in pancreatic ductal adenocarcinoma cells. Cell Oncol (Dordr).

[57] Morin E, Sjöberg E, Tjomsland V (2018). VEGF receptor-2/neuropilin 1 trans-complex formation between endothelial and tumor cells is an independent predictor of pancreatic cancer survival. J Pathol.

[58] Jin X, Pan Y, Wang L (2017). Fructose-1,6-bisphosphatase Inhibits ERK Activation and Bypasses Gemcitabine Resistance in Pancreatic Cancer by Blocking IQGAP1-MAPK Interaction. Cancer Res.

[59] Nie K, Li J, He X (2020). COX6B2 drives metabolic reprogramming toward oxidative phosphorylation to promote metastasis in pancreatic ductal cancer cells. Oncogenesis.

[60] Ratnam NM, Peterson JM, Talbert EE (2017). NF-κB regulates GDF-15 to suppress macrophage surveillance during early tumor development. J Clin Invest.

[61] Drosos Y, Neale G, Ye J (2016). Prox1-Heterozygosis Sensitizes the Pancreas to Oncogenic Kras-Induced Neoplastic Transformation. Neoplasia.

[62] Orozco CA, Martinez-Bosch N, Guerrero PE (2018). Targeting galectin-1 inhibits pancreatic cancer progression by modulating tumor-stroma crosstalk. Proc Natl Acad Sci U S A.

[63] Qian D, Lu Z, Xu Q (2017). Galectin-1-driven upregulation of SDF-1 in pancreatic stellate cells promotes pancreatic cancer metastasis. Cancer Lett.

[64] Zhou LM, Yuan LL, Gao Y (2021). Nucleophosmin 1 overexpression correlates with (18)F-FDG PET/CT metabolic parameters and improves diagnostic accuracy in patients with lung adenocarcinoma. Eur J Nucl Med Mol Imaging.

[65] Buoso E, Masi M, Long A (2020). Ribosomes as a nexus between translation and cancer progression: Focus on ribosomal Receptor for Activated C Kinase 1 (RACK1) in breast cancer. Br J Pharmacol.

[66] Dan H, Liu S, Liu J (2020). RACK1 promotes cancer progression by increasing the M2/M1 macrophage ratio via the NF-κB pathway in oral squamous cell carcinoma. Mol Oncol.

[67] Wang M, Liu J, Zhao Y (2020). Upregulation of METTL14 mediates the elevation of PERP mRNA N(6) adenosine methylation promoting the growth and metastasis of pancreatic cancer. Mol Cancer.

[68] Huang F, Shi Q, Li Y (2018). HER2/EGFR-AKT Signaling Switches TGFβ from Inhibiting Cell Proliferation to Promoting Cell Migration in Breast Cancer. Cancer Res.

[69] Wu KY, Kim S, Liu VM (2021). Function-Blocking RHAMM Peptides Attenuate Fibrosis and Promote Antifibrotic Adipokines in a Bleomycin-Induced Murine Model of Systemic Sclerosis. J Invest Dermatol.

[70] Zhang Z, Huang L, Zhao W (2010). Annexin 1 induced by anti-inflammatory drugs binds to NF-kappaB and inhibits its activation: anticancer effects *in vitro* and *in vivo*. Cancer Res.

[71] Leslie J, Millar BJ, Del Carpio Pons A (2020). FPR-1 is an important regulator of neutrophil recruitment and a tissue-specific driver of pulmonary fibrosis. JCI Insight.

[72] Chen K, Bao Z, Gong W (2017). Regulation of inflammation by members of the formyl-peptide receptor family. J Autoimmun.

[73] Huang C, Li N, Li Z (2017). Tumour-derived Interleukin 35 promotes pancreatic ductal adenocarcinoma cell extravasation and metastasis by inducing ICAM1 expression. Nat Commun.

[74] Wang L, Wang L, Zhang H (2020). AREG mediates the epithelial-mesenchymal transition in pancreatic cancer cells via the EGFR/ERK/NF-κB signalling pathway. Oncol Rep.

[75] Li E, Wei B, Wang X (2020). METTL3 enhances cell adhesion through stabilizing integrin β1 mRNA via an m6A-HuR-dependent mechanism in prostatic carcinoma. Am J Cancer Res.

[76] Markiewski MM, Vadrevu SK, Sharma SK (2017). The Ribosomal Protein S19 Suppresses Antitumor Immune Responses via the Complement C5a Receptor 1. J Immunol.

[77] Ding P, Li L, Li L (2020). C5aR1 is a master regulator in Colorectal Tumorigenesis via Immune modulation. Theranostics.

[78] Mauri P, Scarpa A, Nascimbeni AC (2005). Identification of proteins released by pancreatic cancer cells by multidimensional protein identification technology: a strategy for identification of novel cancer markers. Faseb j.

[79] Roda O, Ortiz-Zapater E, Martínez-Bosch N (2009). Galectin-1 is a novel functional receptor for tissue plasminogen activator in pancreatic cancer. Gastroenterology.

