# Supplementary material for: Single-cell transcriptomics reveals heterogeneous progression and EGFR activation in pancreatic adenosquamous carcinoma
Source: Int J Biol Sci. 2021 Jun 22;17(10):2590–605. doi: 10.7150/ijbs.58886 (PMC8315026; doi:10.7150/ijbs.58886)
Supplement: Supplementary file 1 — Supplementary figures and tables. [file ijbsv17p2590s1.pdf]

Supplementary Figures

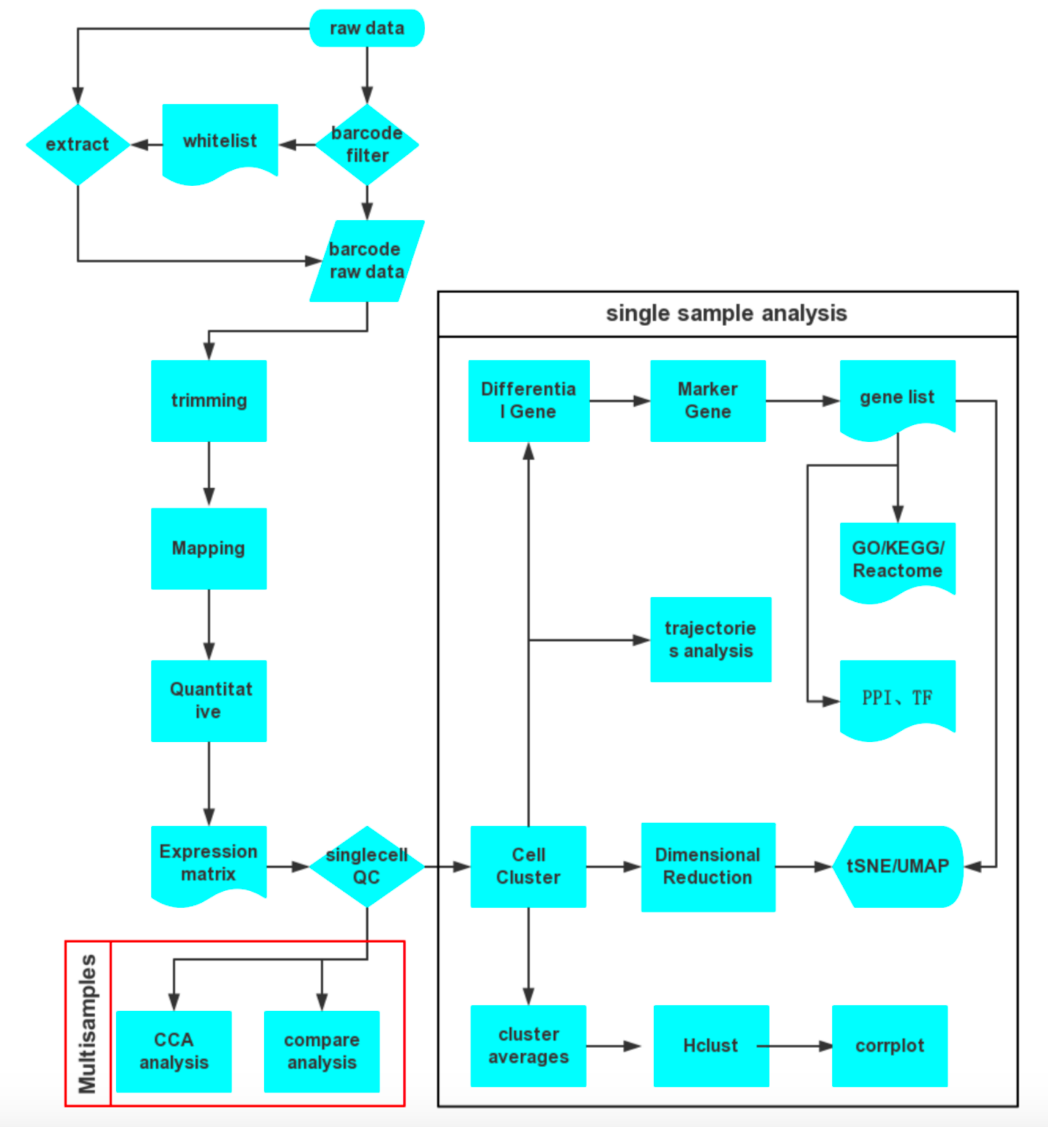

Figure S1. Flowchart of primary bioinformatics analysis for single-cell RNA sequencing.

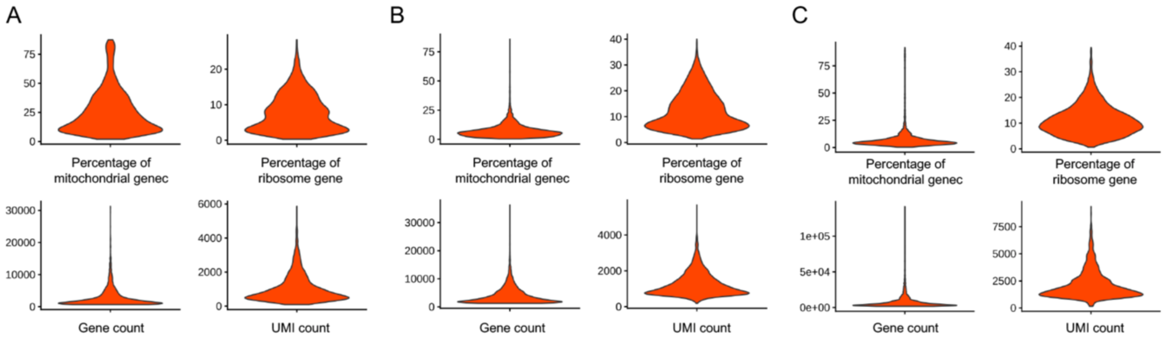

Figure S2. The number of UMIs, genes, and abundance of mitochondrial and ribosomal transcripts of single cells from normal pancreas (A), intraductal papillary mucinous neoplasm (B), and pancreatic adenosquamous carcinoma (C).

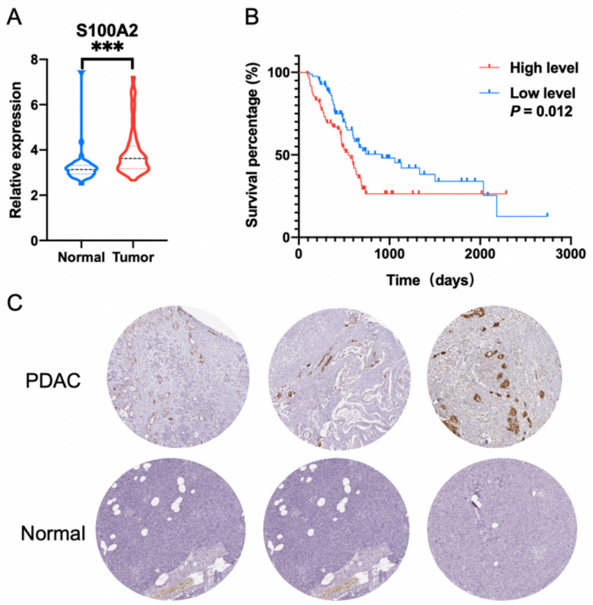

Figure S3. S100A2 is associated with the malignant clinical features of PC. In GSE28735, S100A2 is significantly upregulated in PC tumor tissues (**A**) and is related to poor prognosis in the PDAC cohort of TCGA (**B**). In the Human Protein Atlas, S100A2 was highly expressed in PC tissues but not in the normal pancreas (**C**). *** $P < 0.001$.

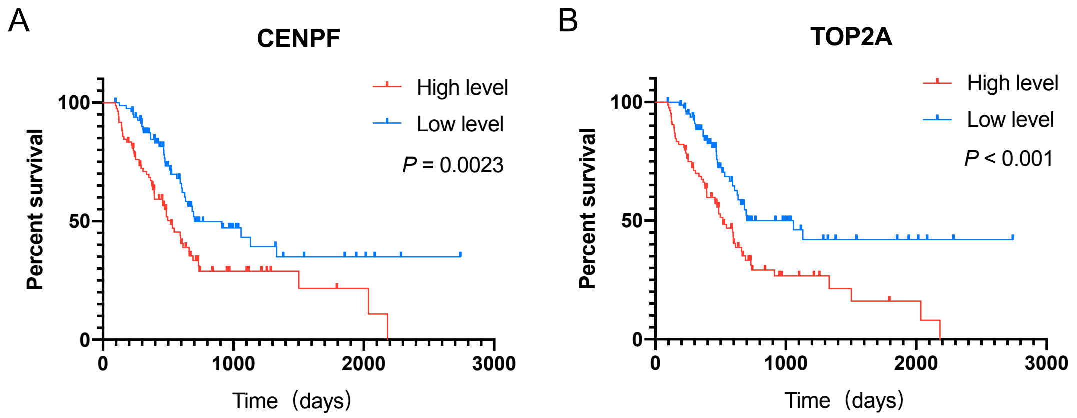

Figure S4. Kaplan-Meier survival analyses indicate that the high levels of CENPF（A）and TOP2A（B）were associated with poor prognosis in the PDAC cohort of TCGA.

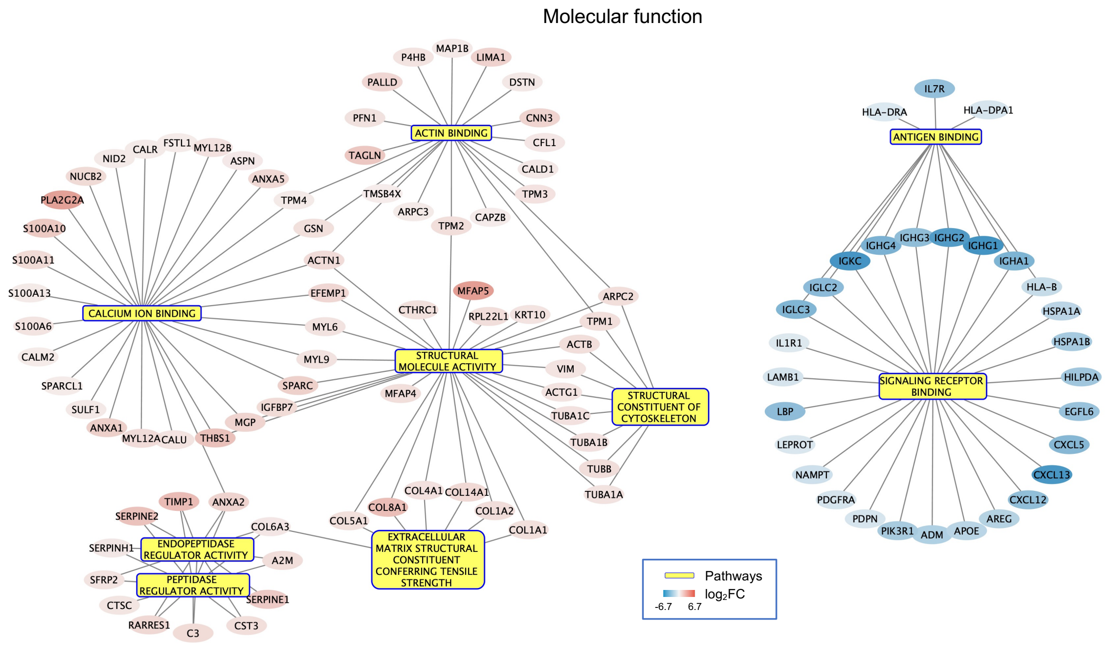

Figure S5. The molecular function of upregulated (left) and downregulated (right) gene sets in cancer-associated fibroblasts compared with fibroblasts.

Cellular component

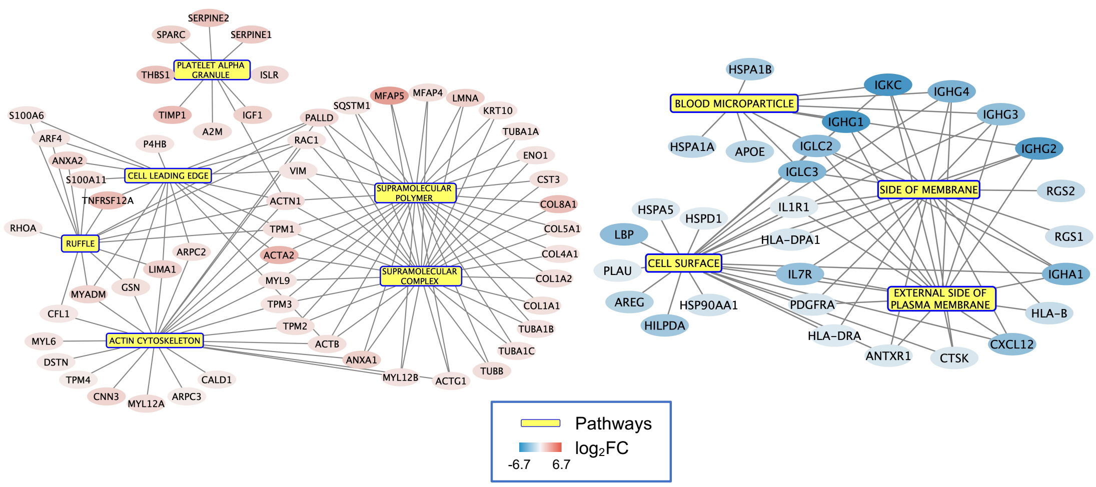

Figure S6. The cellular component of upregulated (left) and downregulated (right) gene sets in cancer-associated fibroblasts compared with fibroblasts.

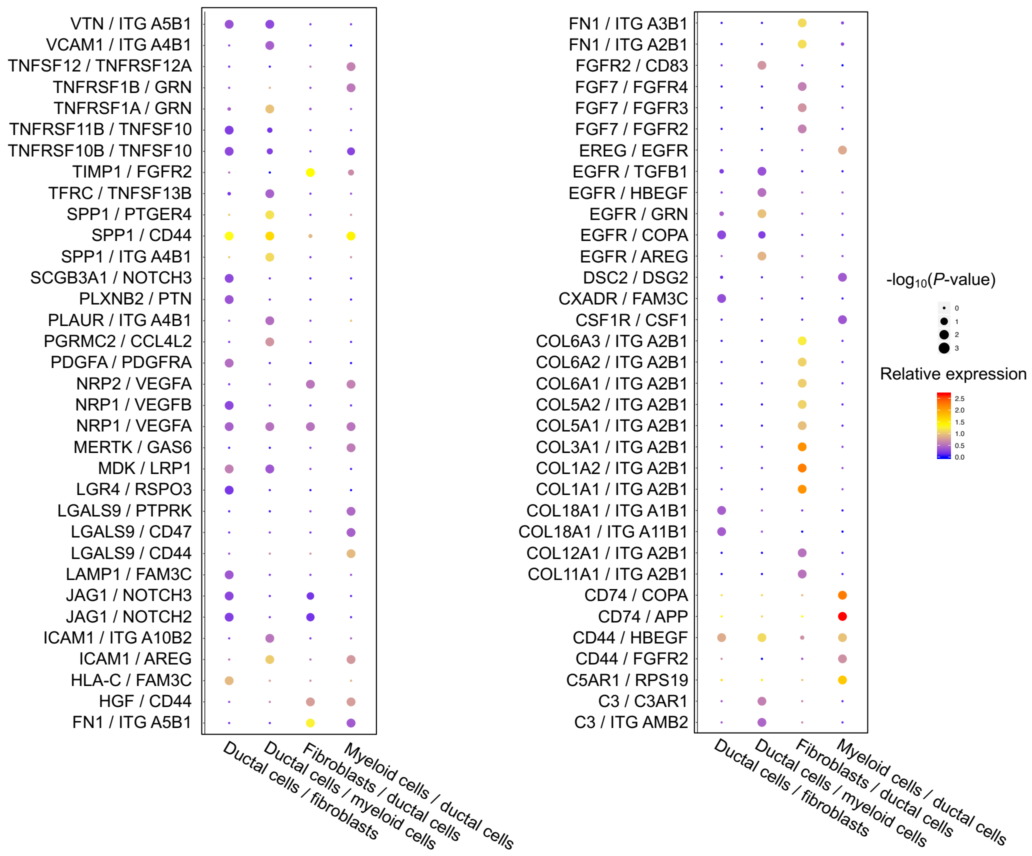

Figure S7. The relative expression level of ligand-receptor of fibroblasts and myeloid cells to pancreatic ductal cells.

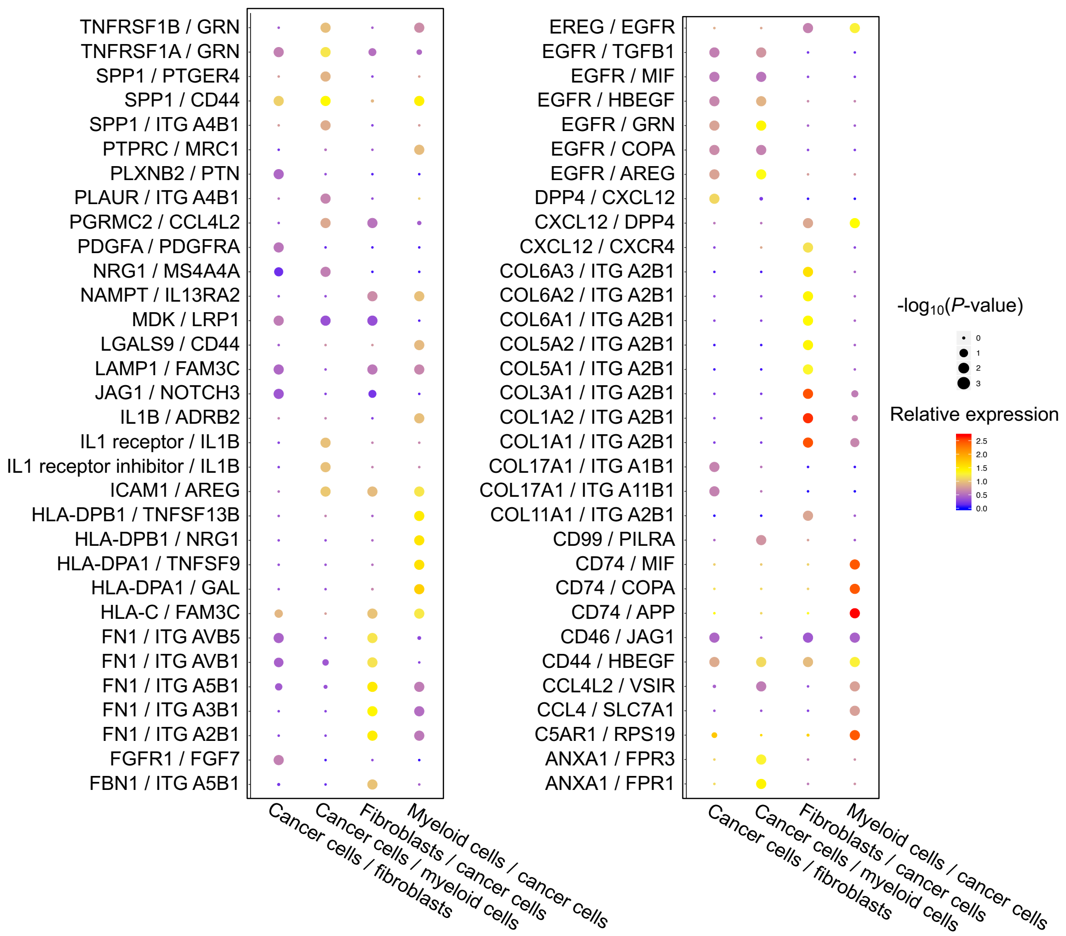

Figure S8. The relative expression level of ligand-receptor of fibroblasts and myeloid cells to pancreatic adenosquamous carcinoma cells.

Supplementary Tables

Table S1. Overall characteristics of sample tissues in this study.

Sample tissues	Gender	Age	Cell number	Median UMIs	Median Genes
Normal pancreas	Male	50	1662	1886	764
Intraductal papillary mucinous neoplasm	Male	74	6151	3107	1074
Pancreatic adenosquamous carcinoma	Male	59	2074	4966	1724

Table S2. Gene markers for identifying cell types.

Cell type	Marker
Myeloid cells	LYZ, C1QA, CD68, CD163, CD1C
Plasma cells	CD79A, JCHAIN, MZB1, IGHG1
B cells	MS4A1, CD79A, CD79B
T cells	CD2, CD3D, TRAC, TRBC2
Fibroblasts	LUM, DCN, COL1A2, COL1A1
Ductal cells	KRT19, KRT7, EPCAM, SOX9, KRT23
Cancer cells	KRT19, KRT7, EPCAM, SOX9, KRT23
Endothelial cells	CDH5, PECAM1, VWF
Acinar cells	PRSS1, CELA3A, CTRB1, CTRB2
Stellate cells	RGS5, ACTA2, ADIRF

Table S3. The top ten gene markers for identifying cell types.

P-value	$log_{10}FC$	Percentage-1	Percentage-2	Adjust *P*-value	Cell cluster	Marker gene
0.00	2.38	0.61	0.27	0.00	Myeloid_cells	IL1B
0.00	2.13	0.76	0.37	0.00	Myeloid_cells	APOC1
0.00	2.09	0.95	0.54	0.00	Myeloid_cells	LYZ
0.00	2.08	0.80	0.30	0.00	Myeloid_cells	C1QB
0.00	2.07	0.96	0.41	0.00	Myeloid_cells	TYROBP
0.00	2.04	0.76	0.22	0.00	Myeloid_cells	C1QC
0.00	1.98	0.90	0.51	0.00	Myeloid_cells	CXCL8
0.00	1.95	0.58	0.23	0.00	Myeloid_cells	EREG
0.00	1.91	0.90	0.30	0.00	Myeloid_cells	FCER1G
0.00	1.89	0.62	0.22	0.00	Myeloid_cells	G0S2
0.00	3.85	1.00	0.59	0.00	Plasma_cells	IGHG1
0.00	3.75	0.98	0.52	0.00	Plasma_cells	IGHG4
0.00	3.70	0.93	0.36	0.00	Plasma_cells	IGHG3
0.00	3.57	1.00	0.65	0.00	Plasma_cells	IGKC
0.00	3.54	0.99	0.55	0.00	Plasma_cells	IGHG2
0.00	2.89	0.77	0.14	0.00	Plasma_cells	JCHAIN
0.00	3.30	0.60	0.19	0.00	Plasma_cells	IGHA2
0.00	3.72	0.93	0.52	0.00	Plasma_cells	IGLC3
0.00	3.74	0.94	0.50	0.00	Plasma_cells	IGHA1
0.00	3.81	0.92	0.52	0.00	Plasma_cells	IGLC2
0.00	2.62	0.88	0.14	0.00	B_cells	MS4A1
0.00	1.77	0.59	0.08	0.00	B_cells	BANK1
0.00	1.71	0.78	0.18	0.00	B_cells	CD79A
0.00	1.60	0.90	0.46	0.00	B_cells	CD52
0.00	1.34	0.34	0.03	0.00	B_cells	VPREB3
0.00	1.29	0.42	0.06	0.00	B_cells	CD79B
0.00	1.27	0.28	0.02	0.00	B_cells	TCL1A
0.00	1.28	0.69	0.29	0.00	B_cells	CD37
0.00	1.26	0.43	0.10	0.00	B_cells	LRMP
0.00	1.67	0.61	0.45	0.00	B_cells	HIST1H4C
0.00	2.10	0.66	0.08	0.00	T_cells	CD2
0.00	1.98	0.65	0.12	0.00	T_cells	TRBC2
0.00	1.92	0.58	0.08	0.00	T_cells	CD3D
0.00	1.66	0.48	0.06	0.00	T_cells	CD7
0.00	1.62	0.52	0.07	0.00	T_cells	TRAC
0.00	2.09	0.40	0.06	0.00	T_cells	GZMA
0.00	1.82	0.44	0.07	0.00	T_cells	TRBC1
0.00	1.61	0.53	0.12	0.00	T_cells	ITM2A
0.00	3.14	0.65	0.25	0.00	T_cells	CCL5
0.00	1.97	0.46	0.11	0.00	T_cells	NKG7
0.00	3.74	0.97	0.34	0.00	Fibroblasts	COL1A1
0.00	3.73	0.99	0.37	0.00	Fibroblasts	COL1A2
0.00	3.58	0.99	0.32	0.00	Fibroblasts	COL3A1
0.00	3.42	0.99	0.32	0.00	Fibroblasts	DCN
0.00	3.19	0.98	0.26	0.00	Fibroblasts	LUM

0.00	3.08	0.79	0.15	0.00	Fibroblasts	POSTN
0.00	2.88	0.93	0.14	0.00	Fibroblasts	SFRP2
0.00	2.87	0.97	0.40	0.00	Fibroblasts	SPARC
0.00	2.49	0.82	0.07	0.00	Fibroblasts	CCDC80
0.00	2.45	0.63	0.21	0.00	Fibroblasts	MGP
0.00	3.22	0.83	0.03	0.00	Ductal_cells	SLC4A4
0.00	2.68	0.83	0.14	0.00	Ductal_cells	CLU
0.00	2.64	0.77	0.09	0.00	Ductal_cells	CLDN4
0.00	2.63	0.74	0.02	0.00	Ductal_cells	CFTR
0.00	2.60	0.65	0.08	0.00	Ductal_cells	MMP7
0.00	2.58	0.87	0.13	0.00	Ductal_cells	MTND1P23
0.00	2.27	0.76	0.16	0.00	Ductal_cells	TM4SF1
0.00	2.25	0.73	0.17	0.00	Ductal_cells	ANXA4
0.00	2.24	0.72	0.11	0.00	Ductal_cells	KRT8
0.00	2.21	0.69	0.08	0.00	Ductal_cells	TACSTD2
0.00	3.59	0.98	0.12	0.00	Cancer_cells	S100A2
0.00	3.20	0.94	0.08	0.00	Cancer_cells	KRT14
0.00	3.15	0.97	0.10	0.00	Cancer_cells	KRT17
0.00	3.04	0.93	0.07	0.00	Cancer_cells	KRT6A
0.00	2.98	0.88	0.07	0.00	Cancer_cells	LY6D
0.00	2.83	0.67	0.06	0.00	Cancer_cells	SPRR1B
0.00	2.80	0.98	0.11	0.00	Cancer_cells	PERP
0.00	2.78	0.96	0.11	0.00	Cancer_cells	SLPI
0.00	2.67	0.73	0.07	0.00	Cancer_cells	PI3
0.00	2.55	0.85	0.04	0.00	Cancer_cells	KRT5
0.00	2.75	0.93	0.16	0.00	Endothelial_cells	SPARCL1
0.00	2.63	0.81	0.02	0.00	Endothelial_cells	PLVAP
0.00	2.28	0.52	0.02	0.00	Endothelial_cells	ACKR1
0.00	2.19	0.64	0.01	0.00	Endothelial_cells	CLDN5
0.00	2.00	0.73	0.07	0.00	Endothelial_cells	HSPG2
0.00	1.86	0.54	0.01	0.00	Endothelial_cells	VWF
0.00	1.89	0.71	0.12	0.00	Endothelial_cells	GNG11
0.00	1.96	0.79	0.17	0.00	Endothelial_cells	PECAM1
0.00	2.02	0.70	0.20	0.00	Endothelial_cells	SPRY1
0.00	1.89	0.30	0.03	0.00	Endothelial_cells	STC1
0.00	4.36	0.81	0.08	0.00	Acinar_cells	REG1B
0.00	3.93	0.92	0.12	0.00	Acinar_cells	SPINK1
0.00	3.86	0.84	0.10	0.00	Acinar_cells	PRSS1
0.00	3.61	0.78	0.05	0.00	Acinar_cells	CTRB1
0.00	3.51	0.74	0.05	0.00	Acinar_cells	PLA2G1B
0.00	4.67	0.92	0.13	0.00	Acinar_cells	CTRB2
0.00	4.60	0.90	0.15	0.00	Acinar_cells	REG3A
0.00	4.39	0.86	0.15	0.00	Acinar_cells	PRSS2
0.00	4.44	0.96	0.22	0.00	Acinar_cells	REG1A
0.00	4.00	0.57	0.34	0.00	Acinar_cells	TAOK1
0.00	4.09	0.96	0.06	0.00	Stellate_cells	RGS5
0.00	2.28	0.73	0.02	0.00	Stellate_cells	NDUFA4L2
0.00	2.17	0.63	0.02	0.00	Stellate_cells	STEAP4

0.00	2.04	0.48	0.02	0.00	Stellate_cells	FABP4
0.00	2.71	0.88	0.09	0.00	Stellate_cells	ADIRF
0.00	2.16	0.59	0.05	0.00	Stellate_cells	IGFBP5
0.00	2.48	0.87	0.17	0.00	Stellate_cells	SPARCL1
0.00	2.13	0.97	0.23	0.00	Stellate_cells	CALD1
0.00	2.84	1.00	0.43	0.00	Stellate_cells	IGFBP7
0.00	2.24	0.69	0.15	0.00	Stellate_cells	CCL2

Percentage-1: Gene expression percentage of each cell cluster.

Percentage-2: Gene expression percentage of all the other cell clusters.

Table S4. The top 20 gene markers for the ductal cells and cancer cells in the initial state of pseudotime trajectory.

Gene	*P*-value	$log_{10}FC$	Percentage-1	Percentage-2	Adjust *P*-value
TFF3	1.40E-62	2.705614568	0.648	0.136	3.14E-58
TCN1	9.32E-93	2.642806011	0.728	0.103	2.10E-88
PIGR	1.26E-108	2.518709188	0.753	0.075	2.83E-104
SCGB3A1	2.99E-26	2.488406285	0.321	0.059	6.71E-22
REG1A	1.41E-48	2.382830109	0.92	0.652	3.16E-44
LYZ	6.04E-44	2.33695798	0.809	0.433	1.36E-39
CRISP3	4.23E-104	2.336295468	0.562	0.023	9.52E-100
SPINK1	3.36E-77	2.326030945	0.914	0.332	7.54E-73
TFF1	3.95E-72	2.243881919	0.519	0.05	8.89E-68
SERPINA1	5.98E-28	1.994262382	0.648	0.298	1.34E-23
MUC5B	1.41E-62	1.88610139	0.525	0.068	3.17E-58
TFF2	9.66E-53	1.849130506	0.34	0.022	2.17E-48
CEACAM6	6.45E-55	1.806832416	0.562	0.102	1.45E-50
CRP	1.74E-34	1.79294195	0.253	0.022	3.92E-30
REG3A	6.56E-06	1.775515723	0.562	0.451	0.14749332
LGALS2	1.31E-75	1.694426402	0.457	0.026	2.94E-71
DUOXA2	2.07E-45	1.650738017	0.364	0.04	4.65E-41
GPX2	1.20E-60	1.585143648	0.63	0.122	2.70E-56
DUOX2	2.61E-49	1.564446197	0.401	0.045	5.86E-45
GC	7.12E-69	1.52032148	0.469	0.035	1.60E-64

Percentage-1: Gene expression percentage of each cancer-cell cluster.

Percentage-2: Gene expression percentage of all the other cancer-cell clusters.

Table S5. The top 20 gene markers for the ductal cells and cancer cells in the first state of pseudotime trajectory.

Gene	*P*-value	$log_{10}FC$	Percentage-1	Percentage-2	Adjust *P*-value
SPRR1B	5.50E-87	3.400432903	0.888	0.244	1.24E-82
S100A7	5.02E-30	3.006358772	0.565	0.191	1.13E-25
S100A8	1.07E-46	2.990666421	0.776	0.272	2.41E-42
S100A9	2.23E-55	2.872422477	0.87	0.357	5.02E-51
PI3	1.28E-48	2.706268566	0.795	0.293	2.87E-44
CSTB	2.06E-59	2.075814438	0.975	0.561	4.63E-55
S100P	4.54E-75	2.058575034	0.863	0.234	1.02E-70
SPRR3	1.16E-26	1.991310658	0.28	0.044	2.61E-22
SAA1	2.89E-48	1.931368985	0.702	0.199	6.49E-44
SPRR2A	1.77E-57	1.861618567	0.385	0.029	3.97E-53
KRT16	2.10E-65	1.840507938	0.832	0.241	4.71E-61
SLPI	1.31E-51	1.789045347	0.981	0.511	2.94E-47
SPRR2D	9.36E-60	1.7537841	0.46	0.047	2.10E-55
LCN2	6.25E-39	1.71971481	0.814	0.328	1.40E-34
IL1RN	2.72E-40	1.701022556	0.727	0.265	6.12E-36
SERPINB3	1.01E-30	1.600366811	0.584	0.204	2.28E-26
CSTA	5.04E-35	1.584867706	0.702	0.268	1.13E-30
DMKN	1.22E-42	1.575187838	0.795	0.319	2.75E-38
RHCG	1.36E-54	1.556660377	0.491	0.068	3.07E-50
LY6D	2.33E-50	1.526358909	0.925	0.35	5.24E-46

Percentage-1: Gene expression percentage of each cancer-cell cluster.

Percentage-2: Gene expression percentage of all the other cancer-cell clusters.

Table S6. The top 20 gene markers for the ductal cells and cancer cells in the second state of pseudotime trajectory.

Gene	*P*-value	$log_{10}FC$	Percentage-1	Percentage-2	Adjust *P*-value
MT1M	0.53598382	2.083149695	0.077	0.116	1
AP000769.1	0.081172703	1.839024709	0.205	0.131	1
TAOK1	0.140673854	1.669892981	0.487	0.424	1
MT1G	0.188255525	1.399301412	0.103	0.195	1
KRT15	0.086572236	1.088061851	0.205	0.13	1
IL1RL1	0.012408123	0.95964239	0.128	0.045	1
MT1L	0.18399315	0.934793071	0.359	0.306	1
UPP1	0.297150527	0.922985738	0.333	0.307	1
TIMP1	0.109511376	0.744154232	0.744	0.692	1
RPL36	9.27E-05	0.708455504	0.974	0.727	1
ARGLU1	0.017571775	0.705195382	0.59	0.48	1
RPS27	0.004023953	0.685576625	1	0.861	1
SPSB1	0.570279599	0.67946803	0.128	0.111	1
RPL39	0.018187224	0.665421695	0.795	0.715	1
RPS5	0.002439554	0.653617766	0.769	0.632	1
FGD6	0.05029753	0.641845943	0.205	0.119	1
RPLP2	0.00026985	0.639408366	0.949	0.815	1
MT1X	0.284520695	0.634246935	0.538	0.48	1
SNHG3	0.832351815	0.624812032	0.154	0.183	1
RPL37	0.00044443	0.624265291	0.974	0.825	1

Percentage-1: Gene expression percentage of each cancer-cell cluster.

Percentage-2: Gene expression percentage of all the other cancer-cell clusters.

Table S7. The top 20 gene markers for the ductal cells and cancer cells in the third state of pseudotime trajectory.

Gene	*P*-value	$log_{10}FC$	Percentage-1	Percentage-2	Adjust *P*-value
SLC4A4	1.69E-160	2.792264599	0.892	0.17	3.79E-156
CFTR	6.48E-127	2.195142955	0.8	0.151	1.46E-122
MTND1P23	1.48E-150	2.065718752	0.948	0.23	3.34E-146
CLU	1.02E-98	1.931802381	0.829	0.319	2.29E-94
CCL2	7.84E-42	1.810293572	0.56	0.256	1.76E-37
MTATP6P1	2.95E-147	1.744124588	0.989	0.82	6.64E-143
TTN	1.05E-52	1.697380226	0.349	0.024	2.35E-48
SFRP5	1.47E-70	1.663622609	0.419	0.016	3.30E-66
MT-ATP8	8.62E-140	1.572952418	0.985	0.881	1.94E-135
MT-CYB	6.89E-145	1.55981171	1	0.977	1.55E-140
MUC6	7.41E-56	1.541064892	0.403	0.044	1.66E-51
AQP1	1.53E-48	1.513947515	0.406	0.073	3.44E-44
GMNN	3.14E-30	1.506978436	0.464	0.233	7.07E-26
DCDC2	8.25E-51	1.500228268	0.401	0.059	1.85E-46
MT-ND3	2.19E-136	1.490502057	0.998	0.917	4.93E-132
KCNQ1OT1	5.84E-12	1.458822121	0.382	0.257	1.31E-07
DEFB1	1.90E-49	1.448901078	0.584	0.216	4.27E-45
MT-ND5	1.14E-128	1.397643604	0.989	0.927	2.56E-124
MT-CO1	2.41E-148	1.394448322	1	0.997	5.42E-144
MT-ND4L	1.33E-104	1.385750121	0.954	0.807	2.98E-100

Percentage-1: Gene expression percentage of each cancer-cell cluster.

Percentage-2: Gene expression percentage of all the other cancer-cell clusters.

Table S8. Gene markers for C1-cancer cells.

Gene	*P*-value	$log_{10}FC$	Percentage-1	Percentage-2	Adjust *P*-value
CENPF	3.28E-53	1.70032237	0.822	0.16	7.37E-49
TOP2A	9.37E-50	1.51949089	0.704	0.096	2.11E-45
UBE2C	3.76E-43	1.35719974	0.778	0.188	8.46E-39
SMC4	2.53E-49	1.31489184	0.97	0.454	5.68E-45
MKI67	1.27E-52	1.30806611	0.83	0.155	2.85E-48
ASPM	3.05E-40	1.3009779	0.63	0.091	6.86E-36
CENPE	3.17E-49	1.24165508	0.689	0.084	7.13E-45
CCNB1	1.06E-42	1.18440137	0.711	0.132	2.38E-38
NUSAP1	7.47E-43	1.15476728	0.681	0.107	1.68E-38
TPX2	1.87E-45	1.14018485	0.733	0.137	4.21E-41
HIST1H4C	6.65E-19	1.08164553	0.756	0.437	1.5E-14
PTTG1	2.34E-33	1.05708473	0.77	0.261	5.25E-29
HMGB2	2.17E-37	1.0550782	0.8	0.264	4.89E-33
TUBA1B	1.98E-36	1.00672079	0.97	0.787	4.45E-32
STMN1	1.15E-40	0.99975397	0.867	0.307	2.58E-36
HMMR	5.53E-43	0.99490088	0.63	0.076	1.24E-38
ANLN	2.91E-43	0.98264457	0.6	0.061	6.54E-39
CDC20	1.86E-46	0.97493887	0.696	0.086	4.18E-42
CKS1B	1.19E-27	0.90994188	0.77	0.332	2.68E-23
CDK1	1.55E-37	0.90697428	0.548	0.063	3.48E-33

Percentage-1: Gene expression percentage of each cancer-cell cluster.

Percentage-2: Gene expression percentage of all the other cancer-cell clusters.

Table S9. Gene markers for C2-cancer cells.

Gene	*P*-value	$\log_{10}$FC	Percentage-1	Percentage-2	Adjust *P*-value
SPRR3	9.21E-19	2.18005336	0.359	0.063	2.07E-14
LCN2	6.92E-25	1.88289219	0.87	0.455	1.55E-20
S100A9	1.49E-08	1.5534445	0.87	0.726	0.00033604
PRSS22	3.45E-38	1.52351538	0.794	0.241	7.75E-34
FTH1	9.34E-29	1.51226957	1	1	2.1E-24
C15orf48	1.21E-16	1.46785418	0.565	0.224	2.72E-12
SPRR2A	2.01E-11	1.45700884	0.344	0.106	4.51E-07
CP	1.68E-14	1.44771317	0.702	0.397	3.78E-10
LGALS3	1.48E-27	1.23568785	0.977	0.817	3.32E-23
LMO7	1.99E-26	1.18504469	0.84	0.442	4.47E-22
SPRR1B	3.58E-13	1.13544635	0.824	0.603	8.04E-09
S100A4	1.22E-14	1.08331916	0.931	0.807	2.74E-10
SAA1	2.41E-07	1.07552668	0.679	0.47	0.0054093
CSTB	1.2E-13	1.06670578	0.985	0.93	2.7E-09
WFDC2	2.98E-11	1.06083138	0.336	0.106	0.00000067
CXCL17	7.88E-22	1.04687899	0.397	0.063	1.77E-17
S100P	1.33E-15	1.0433167	0.878	0.601	2.98E-11
ELF3	2.81E-26	1.01067667	0.855	0.41	6.31E-22
TMPRSS11E	4.84E-13	1.00896176	0.321	0.078	1.09E-08
MXD1	3.52E-25	0.99306069	0.885	0.477	7.91E-21

Percentage-1: Gene expression percentage of each cancer-cell cluster.

Percentage-2: Gene expression percentage of all the other cancer-cell clusters.

Table S10. Gene markers for C3-cancer cells.

Gene	*P*-value	$log_{10}FC$	Percentage-1	Percentage-2	Adjust *P*-value
MMP1	6.46E-20	1.63687734	0.726	0.335	1.45E-15
SERPINE1	2.41E-22	1.23173298	0.803	0.41	5.43E-18
LAMA3	2.44E-25	1.14238776	0.923	0.541	5.47E-21
LAMC2	3.13E-20	1.10227975	0.897	0.553	7.04E-16
LAMB3	6.8E-28	1.02286768	0.991	0.828	1.53E-23
COL17A1	9.74E-23	0.95830365	0.915	0.517	2.19E-18
LGALS1	3.51E-23	0.89772194	1	0.915	7.89E-19
PODXL	2.08E-12	0.85715655	0.624	0.325	4.68E-08
IGFBP6	1.35E-17	0.7849073	0.821	0.449	3.04E-13
ITGB4	1.74E-17	0.7743316	0.838	0.51	3.92E-13
TIMP3	2.05E-07	0.76249224	0.538	0.316	0.00461572
ITGA5	4.22E-13	0.72457194	0.812	0.476	9.49E-09
ITGA2	1.47E-08	0.71872724	0.761	0.498	0.00033087
RAC2	6.99E-18	0.71446029	0.795	0.451	1.57E-13
S100A2	4.57E-17	0.70648227	1	0.971	1.03E-12
IL13RA2	8.33E-16	0.68013956	0.744	0.323	1.87E-11
TGFBI	4.02E-12	0.67130549	0.846	0.587	9.03E-08
SLC2A1	1.44E-10	0.64307929	0.932	0.808	0.00000324
PTHLH	1.95E-12	0.64022903	0.744	0.405	4.39E-08
P3H2	4.9E-16	0.62503402	0.59	0.223	1.1E-11

Percentage-1: Gene expression percentage of each cancer-cell cluster.

Percentage-2: Gene expression percentage of all the other cancer-cell clusters.

Table S11. Gene markers for C4-cancer cells.

Gene	*P*-value	log_{10}FC	Percentage-1	Percentage-2	Adjust *P*-value
CLDN4	8.8597E-08	-1.0179249	0.23	0.6	0.00199175
S100P	6.221E-09	-1.0131698	0.324	0.725	0.00013985
RHCG	8.0262E-07	-0.996839	0.027	0.303	0.01804376
DUSP1	7.8173E-09	-0.7912327	0.541	0.884	0.00017574
GPRC5A	4.3567E-10	-0.7630888	0.108	0.541	9.7943E-06
EMP1	1.3402E-08	-0.7443838	0.297	0.736	0.00030129
LMO7	1.1826E-07	-0.7050998	0.203	0.596	0.00265864
LDLR	1.1924E-10	-0.6501883	0.108	0.56	2.6805E-06
ERO1A	2.7958E-09	-0.6208882	0.216	0.673	6.2852E-05
TM4SF1	5.1335E-08	-0.6202422	0.446	0.831	0.00115406
ITGA2	3.8692E-08	-0.6015087	0.189	0.615	0.00086983
RND3	1.8637E-08	-0.5974277	0.149	0.567	0.00041899
LYPD3	4.2651E-07	-0.5854012	0.027	0.321	0.00958847
UBC	2.6673E-11	-0.56771	0.77	0.987	5.9963E-07
ATP1B1	9.1062E-07	-0.5540433	0.554	0.901	0.02047174
TRIB1	1.7586E-08	-0.5507254	0.054	0.42	0.00039535
DNAJB1	1.1229E-09	-0.5303508	0.284	0.769	2.5243E-05
DNAJA1	4.5786E-08	-0.5292906	0.459	0.866	0.00102932
MAFF	2.2916E-07	-0.5168302	0.135	0.497	0.00515183
P4HB	8.0135E-10	-0.4916018	0.284	0.789	1.8015E-05

Percentage-1: Gene expression percentage of each cancer-cell cluster.

Percentage-2: Gene expression percentage of all the other cancer-cell clusters.

Table S12. Gene markers for C5-cancer cells.

Gene	*P*-value	log_{10}FC	Percentage-1	Percentage-2	Adjust *P*-value
KRT1	2.8162E-18	1.97504208	0.375	0.055	6.3311E-14
AQP3	2.7651E-31	1.93002172	0.931	0.422	6.2162E-27
KRT6B	1.8323E-24	1.60096066	0.861	0.409	4.1192E-20
KRTDAP	3.0651E-09	1.57379438	0.306	0.081	6.8906E-05
DMKN	8.2704E-16	1.56844367	0.847	0.698	1.8593E-11
PI3	5.0309E-07	1.43327872	0.778	0.705	0.01130995
SPRR2D	1.6503E-14	1.40758629	0.542	0.173	3.7102E-10
KRT10	1.4774E-14	1.39825124	0.889	0.575	3.3214E-10
CSTA	5.2357E-11	1.34428691	0.792	0.648	1.177E-06
SPRR1B	1.4589E-13	1.33242041	0.847	0.628	3.2798E-09
IL36G	1.5098E-10	1.29379605	0.292	0.063	3.3942E-06
LYPD3	1.5541E-18	1.27007726	0.639	0.223	3.4937E-14
KLK10	5.7252E-20	1.24922869	0.583	0.147	1.2871E-15
TMEM45A	9.0218E-20	1.24731123	0.764	0.35	2.0282E-15
CALML5	7.2051E-33	1.23529859	0.556	0.061	1.6198E-28
LY6D	1.2626E-20	1.21138293	0.986	0.856	2.8384E-16
KLK11	5.0651E-27	1.17689441	0.639	0.127	1.1387E-22
CLDN4	1.3996E-15	1.17102829	0.875	0.497	3.1464E-11
SPINK5	9.505E-15	1.06186507	0.431	0.098	2.1368E-10
SPRR2E	4.1331E-09	1.05500943	0.264	0.059	9.2916E-05

Percentage-1: Gene expression percentage of each cancer-cell cluster.

Percentage-2: Gene expression percentage of all the other cancer-cell clusters.

Table S13. Gene markers for identifying fibroblasts.

Cell type	Gene marker
Fibroblasts	LUM, DCN, COL1A2, COL1A1
Myofibroblasts	DCN, COL1A1, ACTA2, TAGLN
myofibroblastic CAFs (myCAFs)	PI3, COL1A1, SPRR1B, MMP12
